# Treatment of adult neglected clubfoot with Ponseti method: Consecutive case series of thirty-three feet

**DOI:** 10.1371/journal.pone.0358249

**Published:** 2026-09-15

**Authors:** Davi de Podestá Haje, Helena Côrte Gemayel, Alexandre Vidica Marinho, Paula Beatriz Costa Gonçalves

**Affiliations:** 1 Orthopectus Clinical Center, Brasilia, Brazil; 2 Base Hospital of the Federal District, Brasilia, Brazil; Somaiya Vidyavihar University K J Somaiya College of Engineering, INDIA

## Abstract

**Background:**

There are no prior case series of untreated adults with neglected clubfoot treated with the Ponseti method, and the oldest previously reported case was 30 years old. The objective of this study was to analyze the outcomes of Ponseti treatment in adults with neglected clubfoot.

**Methods:**

Thirty patients were initially treated using the Ponseti method. Twenty-one patients (33 feet; mean age, 34 years; range, 21–53 years) had at least 12 months of follow-up and were included in the final analysis. Additional bone surgery was performed only in feet that were not fully corrected after serial cast changes and Achilles tenotomy. Statistical analysis was used to correlate the following: number of casts; pre-treatment flexibility and foot rotation; age; additional bone surgery beyond tenotomy; **sex**; residual deformity; forefoot abduction before tenotomy; and results according to AOFAS score.

**Results:**

A total of 85% had rigid or mildly flexible feet. A mean of 11.6 casts (3–25) were performed before the Achilles tenotomy. A total of 33% had additional bone surgery after the Ponseti method (casts and Achilles tenotomy). There was a significant improvement in the AOFAS score (32–79) in a mean follow-up of 32 months. More flexible feet required fewer cast changes and showed better outcomes (p < 0.05). Older age and lower forefoot abduction values after the cast period were positively correlated with the need for additional bone surgery (p < 0.05). The percentage of residual deformity was significantly higher in males (p = 0.03).

**Conclusion:**

The method applied proved to be a viable and effective alternative for the treatment of neglected clubfoot in adults.

## Introduction

In underdeveloped countries, there are many neglected adult patients with clubfoot due to obstacles such as poor socioeconomic conditions, including the scarcity of accessible medical resources. Furthermore, the high rate of complications related to surgical treatment eventually discourages physicians and adult patients from starting treatment at an older age [1,2].

Neglected congenital clubfoot in adults can cause pain, functional disability, and physical and psychological suffering [1]. Non-operative treatment using the Ponseti method was initially described for children before walking age. It is based on serial casting, followed by Achilles tenotomy and bracing to prevent recurrences [3–7]. More recently, this method has shown good functional results and low complication rates in children and adolescents [8–10].

The main published studies on the treatment of neglected clubfoot in adults mention correction with surgical methods, such as the use of an Ilizarov external fixator or similar devices, talectomy, and triple arthrodesis [11–14].

More recently, isolated cases of adult congenital clubfoot treated with the Ponseti method have been reported [10,15,16], with no case series in the literature. It is also unknown whether age, sex, foot flexibility, and foot rotation relative to the leg are prognostic factors for treatment in adults. In addition, some information about results in adults with neglected clubfoot treated with Ponseti method are unknown such as: a) if it is possible to treat consecutive cases; b) age limits, the mean number of casts, prognosis according to gender and foot flexibility, the need for additional bone surgery after doing the Ponseti casts and the tenotomy, the possible complications, risk of recurrences and importance of brace protocol after treatment.

The objective of this study is to report an unprecedented series of consecutive cases of adult patients with previously untreated congenital clubfoot treated using the Ponseti method. Other minor objectives are to assess whether the following factors influenced the treatment outcome: age, sex, flexibility, foot rotation relative to the leg, and brace adherence.

## Methods

This retrospective, consecutive case series was conducted at a tertiary hospital from January 2019 to August 2024, with medical records analyzed. The medical records were assessed on January 09^th^ 2024, and again on August 09^th^ 2024. The study was approved by the Institutional Review Board. This study was performed in line with the principles of the Declaration of Helsinki. Approval was granted by the Ethics Committee number 53452216.9.3001.5463. All patients included in this manuscript have given written informed consent (as outlined in PLOS consent form) to publish these case details, including the images in Figures and Videos (S1 Videos in S1 File).

Forty-three consecutive adult patients with congenital clubfoot who had not received prior treatment were evaluated. Five patients were excluded due to conditions associated with a substantially increased risk of thrombosis (previous personal thrombosis or significant varicose veins, n = 2) or arterial disease (diabetes mellitus or chronic smoking, n = 3). Although such patients could theoretically be managed with prophylactic anticoagulation, the treating surgeon considered these risk factors incompatible with the prolonged casting period required by this protocol and therefore excluded them for safety reasons. Patients excluded from the sample were not treated with other methods. Two patients declined the proposed treatment.

Only adult patients were selected, that is, those older than adolescence (21 years and 0 days of age), as stipulated by the American Academy of Pediatrics [17], and none had reported prior treatment.

**Fig 1:**
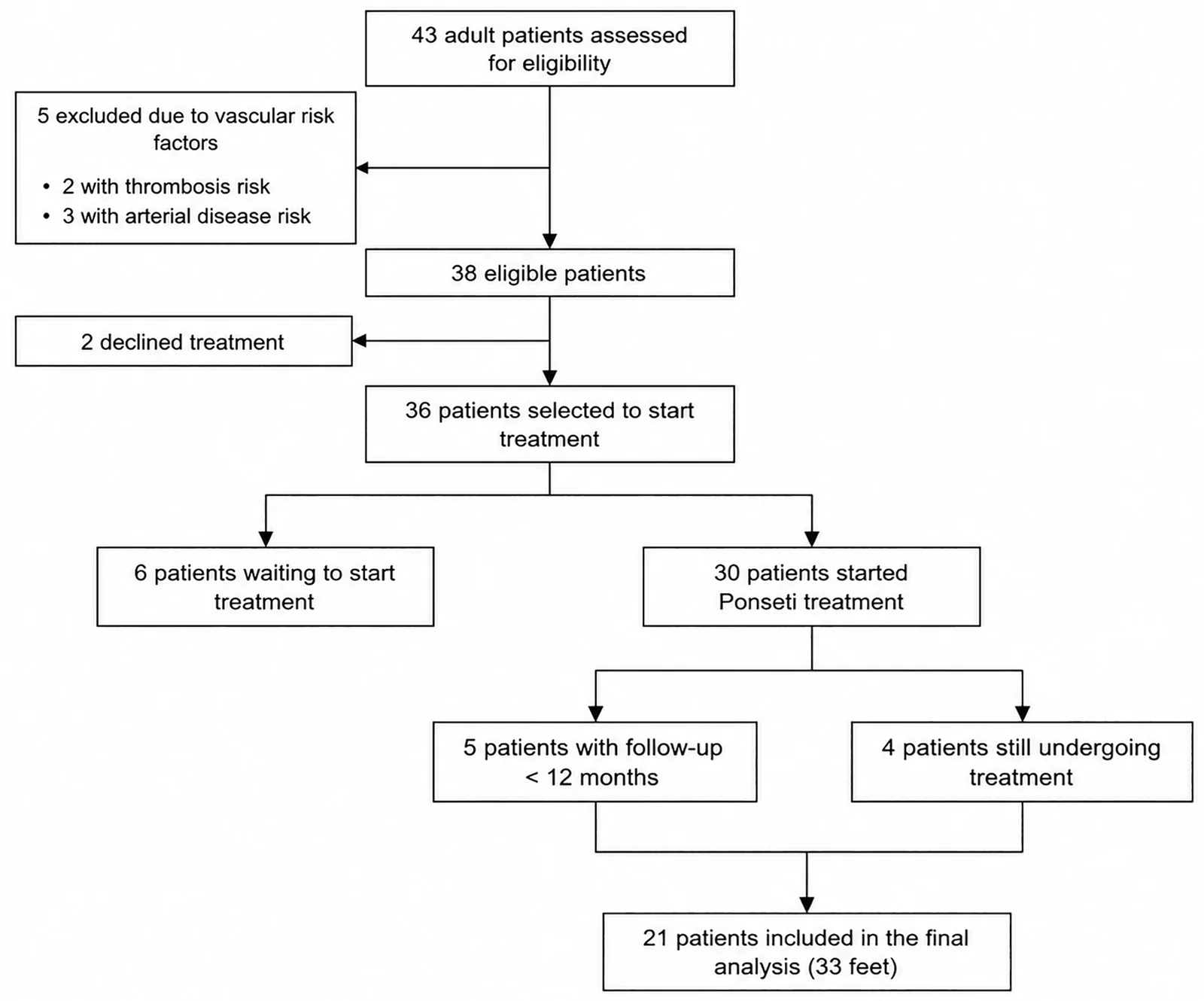
Flowchart illustrating the patient selection and inclusion process.

Out of those, 36 patients were selected to start treatment by applying the Ponseti method, with six waiting to start treatment and nine being excluded from results analysis due to follow-up time of less than 12 months after plaster cast removal (n = 5) or undergoing treatment (n = 4), totaling 21 patients (33 feet). The selection process is shown in Fig 1.

The following were assessed when obtaining the patient’s medical history: concomitant diseases, reasons for not undergoing prior childhood treatment, family history, current symptoms, and daily limitations.

**Fig 2:**
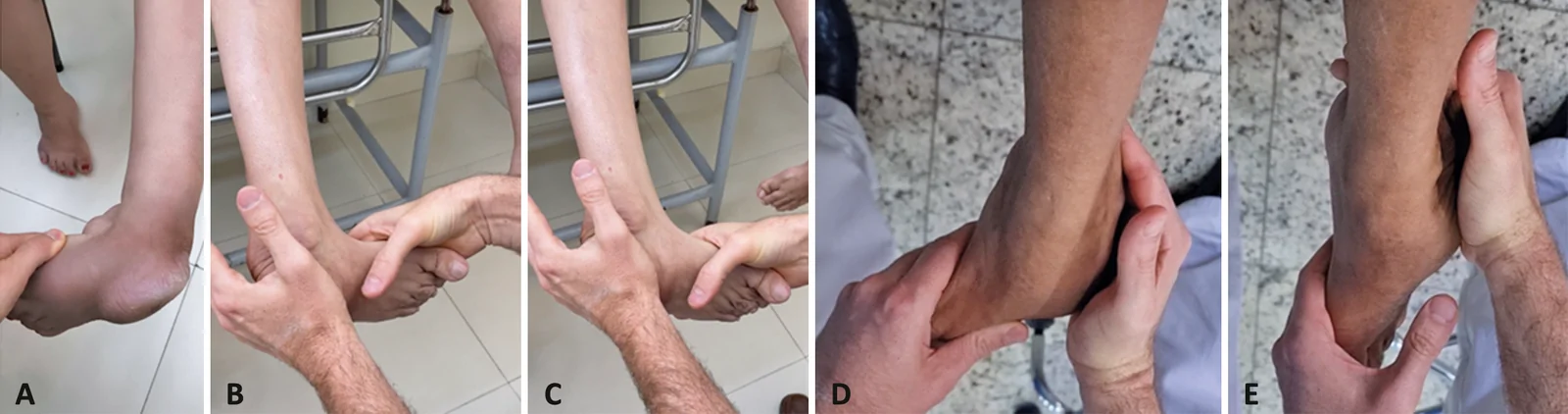
Flexibility test. Correction maneuver: performed passively, forcing correction of cavus and bringing the foot into abduction, considering rigid (**A**), mild flexibility, when there was some correction of cavus and adduction (**B, C**), moderate flexibility, when cavus was significantly reduced, and the adduction was corrected by more than 30° (**D, E**).

**Fig 3:**
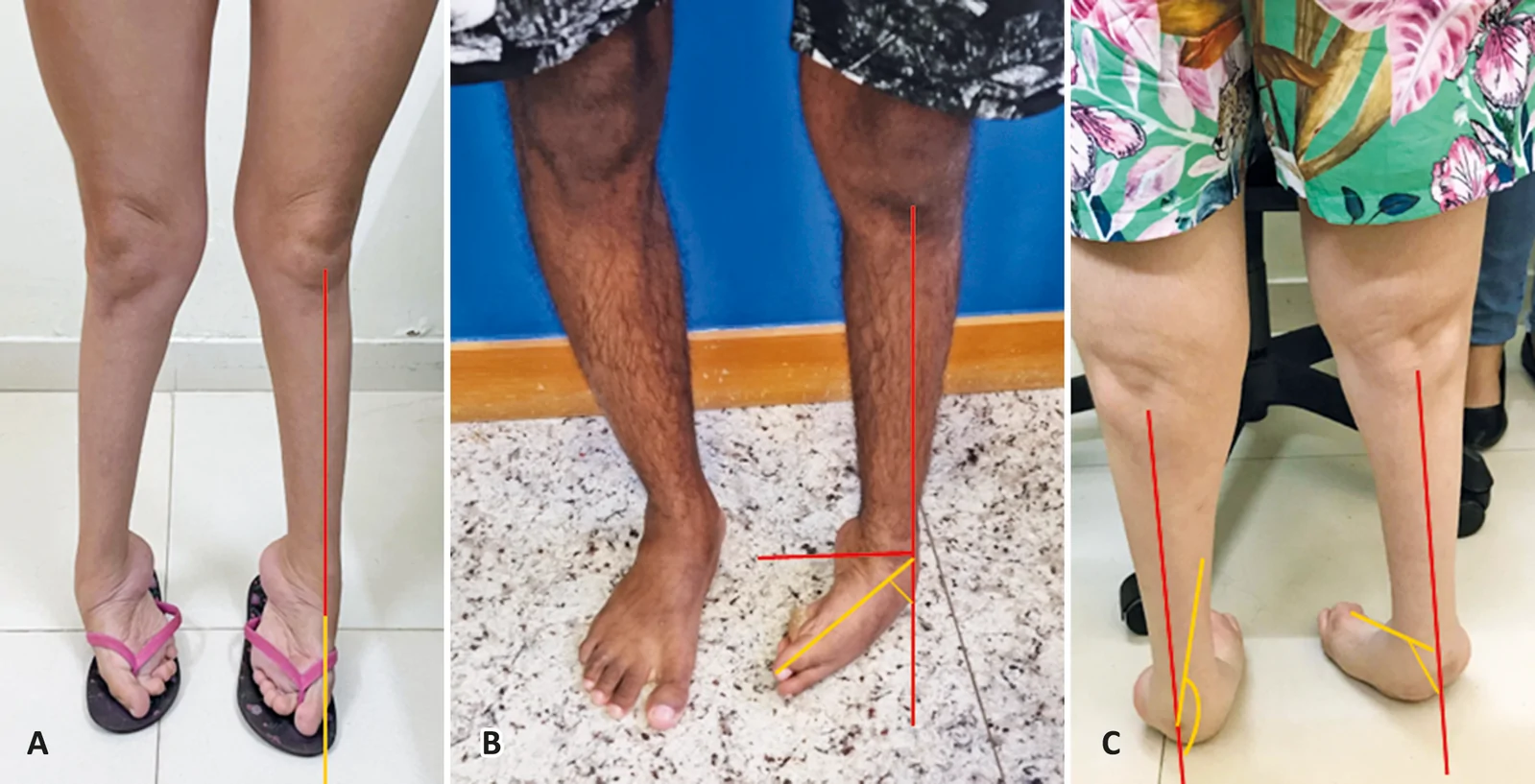
Different rotation patterns of the foot in relation to the tibia (grade 1: between 0° and 45º (A); grade 2: 45° and 90° (B), and grade 3: 90° and 180°(C). Foot rotation was measured as the angle formed between the longitudinal axis of the medial border of the forefoot and the longitudinal axis of the tibia (from the center of the ankle to the tibial tuberosity) on a frontal weight-bearing photograph obtained with the patient standing in a static position, with the patellae facing directly forward and without lower-limb rotation.

**Fig 4:**
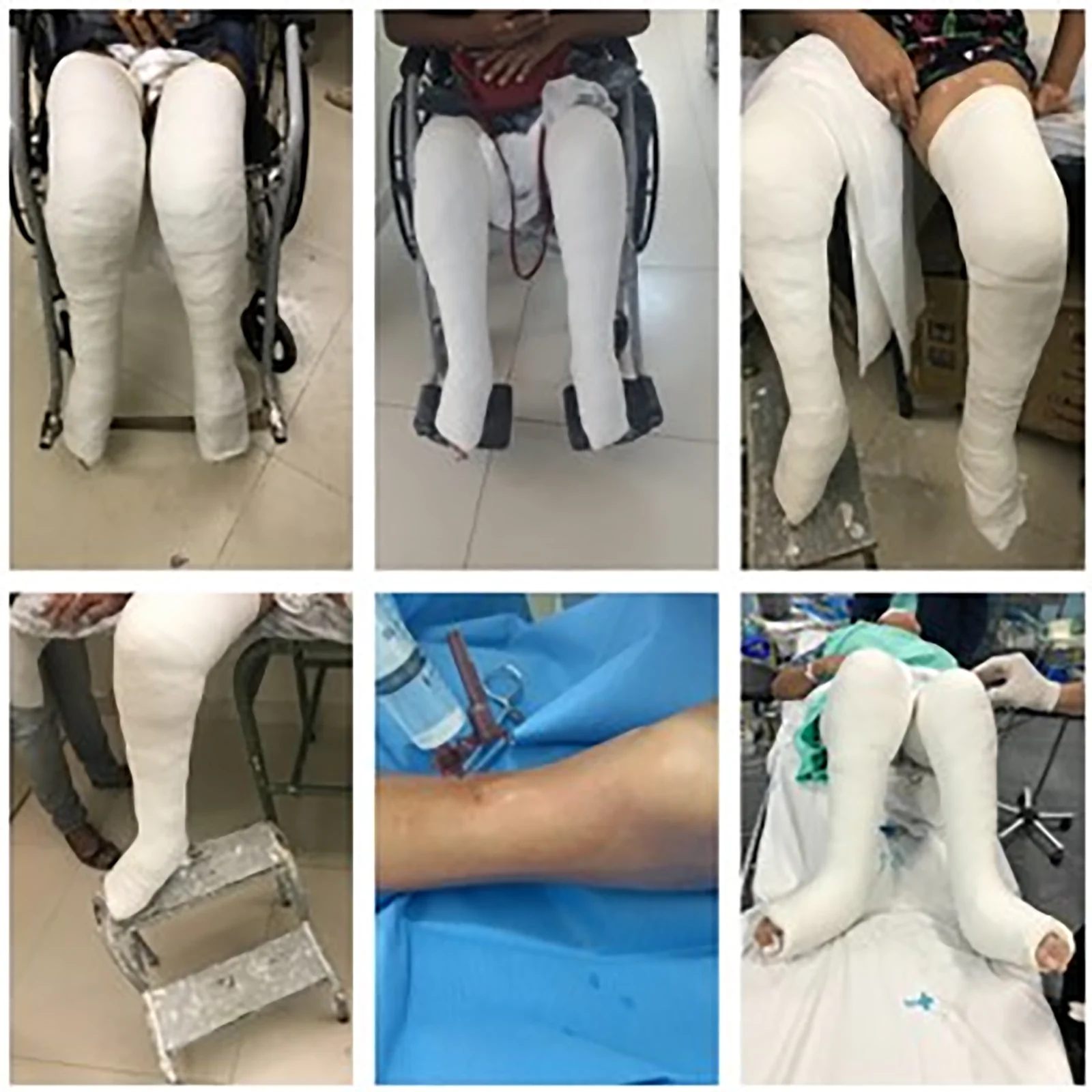
Weekly consecutive casts were done until maximum forefoot abduction (A to D), followed by local anesthesia (E) and sedation. Cast after Achilles tenotomy (**F**).

On physical examination, the following were analyzed: 1) presence of calluses; 2) flexibility (Fig 2); 3) degree of foot rotation relative to the leg (Fig 3); 4) AOFAS score (before and after treatment) and final overall outcomes (Table 1), being a good to excellent result considered as an adequate clinical outcome; 5) degree of foot abduction before tenotomy.

**Table 1:** General outcome: AOFAS’s score questions before and after treatment, and results according to AOFAS score at the last follow-up.

Pain (40 points)	None (+40); mild, occasional (+30); moderate, daily (+20), severe, almost present (+0)
**Function (50 points)**	Activity limitations, support requirements: no limitations, no support (+10); No limitation of daily activities, limitations of recreational activities, no support (+7); Limited daily and recreational activities, cane (+4); Severe limitation of daily and recreational activities, walker, crutches, wheelchair, brace (+0)
	Maximum walking distance, blocks: Greater than six (+5); Four- six (+4); One-three (+2); Less than one (+0)
	Walking surfaces: No difficulty on any surface (+5); Some difficulty on uneven terrain, stairs, inclines, ladders (+3); Severe difficulty on uneven terrain, stairs, inclines, ladders (+0)
	Gait abnormality: None, slight +8); Obvious (+4); Marked (+0)
	Sagittal motion (flexion plus extension): Normal or mild restriction (30° or more) (+8); Moderate restriction (15° - 29°) (+4); Severe restriction (less than 15°) (+0)
	Hindfoot motion (inversion plus eversion): Normal or mild restriction (75% − 100% normal) (+6); Moderate restriction (25% − 74% normal) (+3); Marked restriction (less than 25% of normal) (+0)
	Ankle-hindfoot stability (anteroposterior, varus-valgus): Stable (+8); Definitely unstable (+0)
**Alignment (10 points)**	Good, plantigrade foot, ankle-hindfoot well aligned (+10); Fair, plantigrade foot, some degree of ankle-hindfoot malalignment observed, no symptoms (+5); Poor, nonplantigrade foot, severe malalignment, symptoms (+0)
**Excellent**	80 to 100 points
**Very good**	70-79
**Good**	60-69
**Regular**	50-59
**Poor**	< 50

Pre- and post-treatment radiographs of the feet and ankle were taken in the anteroposterior and profile (weight-bearing), and oblique views, and the following angles were verified by three evaluators in RadiAnt® software: a) tibiocalcaneal angle (profile view of the ankle; 90°-120°); b) calcaneal pitch (8°-30°), c) Meary-Tomeno angle (0° ± 4°) and d) lateral talocalcaneal angle (25°-40°). Bone deformity precluded angle measurements before treatment, except for the tibiocalcaneal angle. Patients were also photographed before treatment and at the end of follow-up, with anterior, posterior, and lateral views of the lower limbs in weight-bearing position. Rivaroxaban (10 mg/day) was prescribed for overweight patients (n = 1) and those older than 50 years (n = 3). In these cases, anticoagulation was considered a relative indication and prescribed based on individualized risk assessment.

During the treatment, serial above-the-knee casts were instrumented sequentially using the Ponseti methodology [9,10,18,19] with intervals of seven days, until all deformities were corrected, except for the equinus, to achieve a forefoot abduction of 30° to 50°. Bilateral cases were treated simultaneously. All patients were asked about pain during treatment and were recommended to take an oral analgesic only when needed. Preferably, 30 mg of oral codeine was taken 30 minutes before the cast change. To avoid excessive muscle and bone weakness, the maximum number of cast changes accepted was 15 (except in one patient who asked to continue cast changes).

Then, tenotomy of the Achilles tendon was performed in the operating room with the patient under sedation associated with local anesthesia in the first five patients. In the subsequent patients, the procedure was performed under spinal anesthesia. The only exception was the patient with myelomeningocele, who underwent percutaneous Achilles tenotomy under local anesthesia without sedation in the outpatient clinic. Fig 4 shows weekly consecutive casts performed until maximal forefoot abduction, followed by local anesthesia and sedation, and the cast after Achilles tenotomy.

At the same time, in cases where complete correction of the cavus, varus, and adduction deformities was not observed after the initial period of cast and Achilles tenotomy, it was necessary to associate an “a la carte” surgical procedure for the correction of residual deformities.

**Fig 5:**
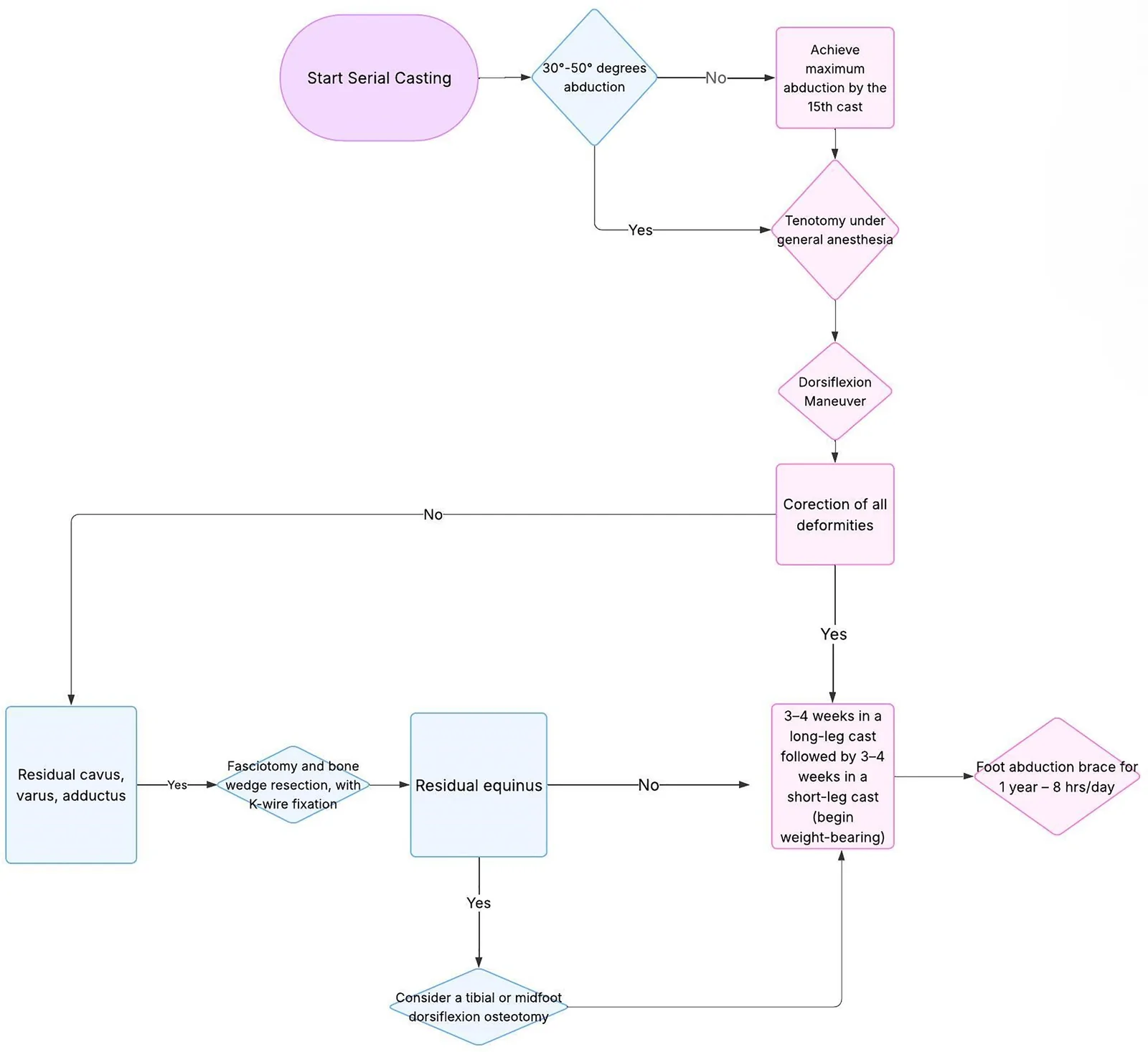
Treatment flowchart illustrating the management pathway for adult neglected clubfoot treated with the Ponseti method.

The surgical procedure was followed by a long-leg cast for three weeks. After this period, a short cast was instrumented with weight-bearing for three to six weeks, and, finally, the double abduction orthosis, with the feet in 40° of external rotation, was worn for eight hours/day for one year (Fig 5). Next, physiotherapy was prescribed, including stretching, strengthening, proprioception, and gait training.

In the physical examination at the end of the follow-up, residual deformities (analyzed hindfoot and forefoot alignment, abnormal cavus or flatfoot, and residual equinus) or calluses and the sole of the foot with thin or sensitive skin were investigated, and the passive mobility of the tibiotarsal and subtalar joints was assessed with a goniometer. A satisfactory outcome was defined as a plantigrade foot that allowed the patient to bear weight and wear tennis shoes. At the end of follow-up, patients were asked whether they would repeat the treatment**.** In patients with bilateral clubfoot, outcome analyses were performed per foot rather than per patient.

Cast adherence and the possibility of relapse were assessed during follow-up.

All clinical assessments, treatment decisions, and surgical procedures were performed by the senior author.

### Statistical analysis

Statistical analysis was used to correlate the following variables: number of casts vs flexibility; number of casts vs pre-treatment foot rotation; number of casts vs age; age vs need for additional bone surgery beyond tenotomy; sex vs need for additional bone surgery; sex vs residual deformity; age vs residual deformity; forefoot abduction before tenotomy vs need for additional bone surgery beyond tenotomy; flexibility vs results; results vs need for additional bone surgery beyond tenotomy, compared with results vs tenotomy alone; AOFAS score pre-treatment vs post-treatment.

For categorical variables, the frequency (n) and percentage (%) for each category were presented; for numerical variables, the mean, standard deviation, median, minimum, and maximum were presented.

For associations between categorical variables, the chi-square (χ2) test was used; for interactions between categorical and numerical variables, the analysis of variance (ANOVA) with Bonferroni’s post hoc test was applied. Correlations were assessed using Spearman’s rank correlation coefficient, and pre- and post-treatment AOFAS scores were compared using the Wilcoxon signed-rank test. A significance level of p ≤ 0.05 was used in all analyses.

## Results

Table 2 shows information collected in the anamnesis of the 43 patients who were initially evaluated.

**Table 2:** Patient medical history about all the 43 consecutive patients initially evaluated.

Patients	Number (N)	Percentage
Patients without prior treatment due to socioeconomic difficulties	43	100%
Patients that sought medical care in adulthood before coming to our service	30	71%
Patients not accepting surgical risks or not provided treatment option on other medical services	17	39%
Patients seeking less aggressive and risky treatment using the Ponseti method	43	100%
Patients with family history of clubfoot	5	11%
Patients with history of foot/feet injury or infection	8	18%
Patients reporting pain on exertion in their feet	43	100%
Patients reporting work activities requiring prolonged time in orthostasis and worsening of pain during this period	12	28%
Impossibility of wearing closed shoes	37	86%
Weight bearing mainly on the dorsolateral region of the feet, with formation of cutaneous calluses in the region.	43	100%

Table 3 presents data from the 21 patients who were treated and met the follow-up time criteria, 62% were male and 38% female, with a mean age of 34 years old (21–53). The deformity was bilateral in 57%, 85% had mild or no flexibility, and 67% had grade 2 foot rotation (45°-90°). The mean cast changes before the Achilles tenotomy were 11.6 casts (3–25). The mean number of casts before tenotomy in those with grade 1 foot rotation was the lowest (only 3).

**Table 3:** Information about all 21 consecutive patients treated with follow-up greater than 12 months.

Patient	Sex	Side	Flexibility	Foot rotation^1^	Casts number^2^	Foot abduction^2^	Associated bone surgery to Achilles tenotomy	Follow-up^3^ (months)	Residual deformity in the last follow-up	Partial recurrence/brace protocol adherence	AOFAS pretreatment	AOFAS posttreatment results^4^
1	F	B	Mild	R:2; L:2	R:15; L:15	R: 45°; L: 40°	No	61	No	No/ Yes	36	97-excellent
2	F	Left	Rigid	3	12	25°	No	55	No, but mild overcorrection	No/ No	8	81-excellent
3	F	B	Rigid with rigid claw toes	R:3; L:3	R:25; L:15	R: 15°; L:30°	No	39	No	No/ Yes	10	80-excellent;
4	M	B	Rigid	R:2; L:2	R:15; L15	R: 10°; L:5°	Yes	35	Mild cavus, varus	No/ Yes	47	79-very good
5	M	B	Rigid	R:2; L:2	R:15; L15	R: 10º; L:10°	Yes	55	Mild cavus, varus	No/ Yes	36	78-very good
6	M	B	Rigid	R:2; L:2	R: 14; L14	R: 40°; L:40°	No	21	Mild cavus, varus	Yes (left foot)/ Yes	50	L77-very good, R82-excellent
7	M	Left	Rigid	2	15	5°	Yes	45	Moderate cavus, varus, adduction	No/ No	26	57-regular
8	M	Left	Moderate	2	3	45°	No	27	No	No/ Yes	36	93-excellent
9	M	Left	Moderate	1	3	50°	No	20	No	No/ Yes	47	81-excellent
10	F	B	Mild	R:2; L:2	R:14; L14	R: 30; L:40°	No	19	L: Mild varus R: No	Yes (left foot)/ Yes	19	L58-regular, R83-excellent
11	M	B	Mild	R:1; L:2	R: 14; L14	R: 30°; L:40°	No	19	L: Moderate varus R: Mild varus	Yes (left foot)/ No	43	L59-regular, R79-very good
12	M	B	Mild	R:2; L:2	R: 8; L: 8	R: 45°; L:45°	No	49	No	No/ Yes	20	81-excellent
13	M	Right	Rigid	2	3	15°	Yes	31	Mild cavus, varus	No/ Yes	19	74-very good
14	M	Left	Rigid	2	15	30°	Yes	33	Mild cavus	No/ No	17	75-very good
15	F	B	Moderate	R:1; L:1	3	R: 50° L: 50º	No	23	L: Mild equinus R No	Yes (left foot)/ No	43	L66-good, R81-excellent
16	M	Right	Mild	2	R: 8; L: 8	55°	No	42	No	No/ Yes	39	93-excellent
17	M	Right	Mild	2	10	40°	No	39	No	No/ No	43	78-very good
18	F	Left	Mild	2	7	40º	No	14	No	No/ Yes	50	90-excellent
19	M	B	Rigid	R:1; L:1	10	R:30°; L:30°	Yes, bilateral	13	No	No/ Yes	39	73-very good
20	F	B	Rigid	R:2; L:2	15	R:20°; L20°	Yes, bilateral	13	No	No/ Yes	12	70-very good
21	F	B	R: Mild; L: Moderate	R:3; L:3	13	R:25º; L:35°	No	12	L: mild equinus, varus; R: No	No/ Yes	17	R75-very good; R80-excellent
**Analyses**	**F:38% M:62%**	**R:14% L:29% B:57%**	**Mild:36% Mod.:15**% **Rigid:49%**	**1: 18%** **2: 67%** **3: 15%**	**11.6 (3-** **25)**	**Mean: 32º (5°-55º)**	**Yes: 33%**	**32 (12-61)**	**No:58%** **Mild:36% Moderate: 6%;**	**Recurrences: 12%/ Yes:72% No: 28%**	**32 (8.0-50.0)**	**79 (57-97.0)** **E: 45%; VG: 42%; G: 3%; Re: 10%**

1 – Grade 1: from 0° to 45°; Grade 2: 45° to 90°; and Grade 3: 90° to 180°; 2 – before Achilles tenotomy or surgery; 3 – after taking out the last cast; 4 – posttreatment results according to Table 1; P.

The Achilles tenotomy was performed as the only surgical procedure in 22 feet (67%).

Additional bone surgery was required in 11 feet (33%), belonging to seven patients (four with bilateral involvement), to correct residual deformity after the Ponseti method: a) percutaneous fasciotomy in association to a lateral wedge resection in the calcaneus-cuboid and subtalar joint, which was fixed with Kirschner wires (n = 9); b) percutaneous Dwyer osteotomy associated with a valgization osteotomy of the distal tibia (n = 1); and c) dorsiflexor osteotomy was performed in the distal tibia (n = 1) because the equinus was not fully corrected after Achilles tenotomy. None of the patients with foot rotation grade 3 needed bone surgery.

The passive mobility observed in those treated exclusively by the Ponseti method without bone surgery was the following mean joint amplitudes in the last evaluation: plantar flexion 14° (7°-20°), dorsiflexion 10° (5°-15°), inversion 7° (5°-10°), and eversion up to 5° (0°-10°).

In those who underwent additional surgery on bone parts, the passive range of motion at the last assessment was: 10° plantar flexion (0°-20°), 5° dorsiflexion (0°-10°), and inversion and eversion up to 0°.

**Fig 6:**
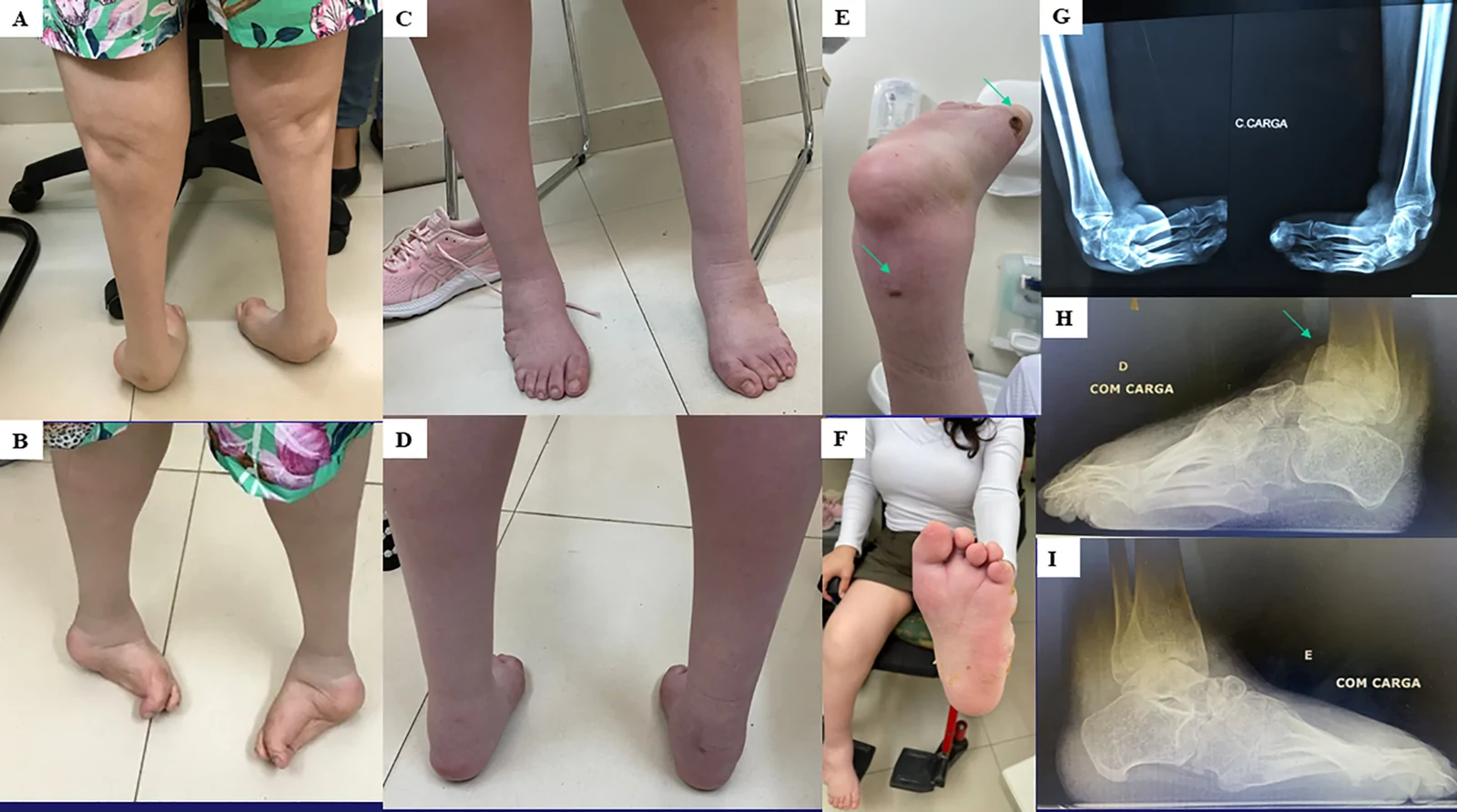
Clinical appearance before treatment. Rigid clubfeet with rigid claw toes in a 38-year-old patient (Patient 3 – Table 3) (**A, B)**. Clinical appearance after treatment. **(C, D).** The arrow indicates the tenotomy scar **(E).** The patient has plantigrade feet. Radiographs before treatment **(G)**. Radiographs after treatment. In the right foot, there was a fracture of the distal anterior tibia after the dorsiflexion maneuver to correct the equinus **(H, I).**

During treatment, the main complications were: skin lesions (n = 3) and an impaction fracture of the anterior region of the tibial pilon during the dorsiflexion maneuver to correct equinus (Fig 6 illustrates the right foot of patient 3; a similar pilon impaction fracture was also observed in the left foot of patient 20). All patients reported the need to use painkillers immediately before and just after the casting. The most painful moment was during cast molding. Although some stiffness and discomfort were reported in the knees immediately after the last cast, all patients regained their pre-treatment range of motion and reported no pain at the last follow-up.

A total of 62% (n = 13) of patients had access to specialized physiotherapy after cast removal.

Recurrences were partial and mild, occurred in 12% (4 feet), and 50% (n = 2) did not follow the brace protocol (foot abduction brace). A total of 83% of feet without relapse were among patients who reported using the brace as prescribed. At the last follow-up, 19 of 33 feet (57.6%) had no residual deformity, whereas 14 feet (42.4%) had residual deformity. Residual deformity was mild in 12 feet (36.4%) and moderate in two feet (6.1%).

**Fig 7:**
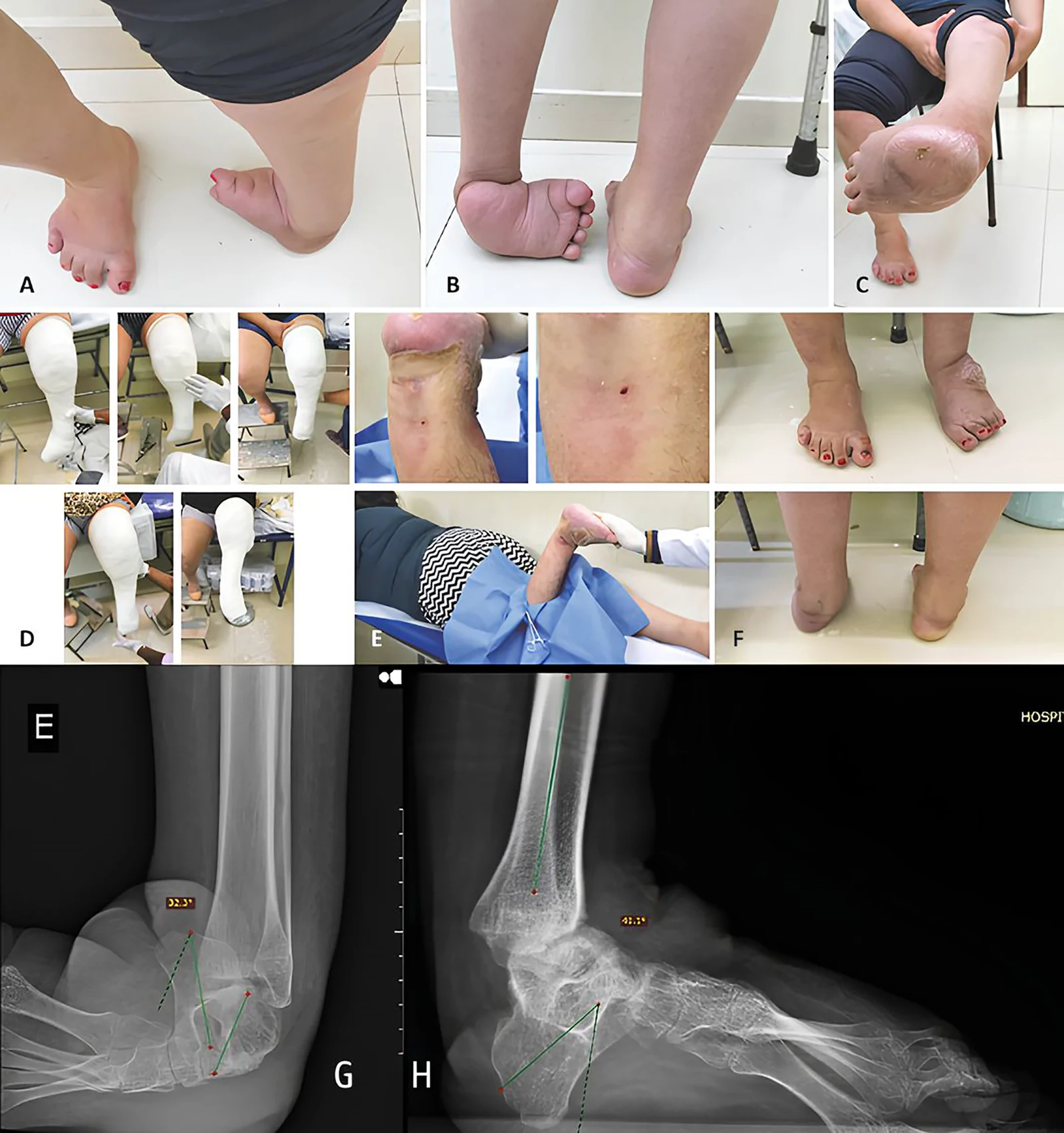
Clinical aspect of a rigid clubfoot of a myelomeningocele patient with obesity and recurrent ulcers before treatment (A, B, C). Sequential casts until 25° foot abduction (**D**). All the treatment was performed on an outpatient basis, even the Achilles tenotomy (with a minimum scar) and dorsiflexion maneuver to correct equinus (**E**). Final clinical aspect, with some degree of overcorrection (**F**). Radiographic appearance after treatment. Tibiocalcaneal angles are increasing due to equinus correction (**G, H**).

The mean value of the radiographic angles was: a) tibiocalcaneal pre-treatment 46.75° ± 23.01° (right) and 38.86° ± 21.68° (left), and in the post-treatment were 101.3° ± 17.10° (right) and 98.32° ± 23.6° (left); b) Meary-Tomeno 17.67° ± 12.01° (right) and 23° ± 10.76° (left); c) calcaneal pitch 17.13° ± 8.77° (right) and 19.42° ± 13.77° (left); and d) talocalcaneal angle 19.5° ± 6.43° (right) and 23.25° ± 13.63° (left). The tibiocalcaneal angle is shown in Fig 7 (patient 3 of Table 3).

Table 4 presents a summary of correlations among clinical variables, treatment factors, and outcomes in adult congenital clubfoot treated with the Ponseti method.

**Table 4:** Correlation analyses between clinical variables and treatment outcomes.

Variable	Comparison/ Correlation Tested	Finding	p-value	Significance
Number of casts	Age and pretreatment foot rotation	No statistically significant correlation	0.54	Not significant
Age	Need for additional bone surgery	Older age correlated with a higher likelihood of additional surgery	0.04	Significant
Sex	Need for additional bone surgery	No significant association	0.25	Not significant
Sex	Percentage of residual deformity	Males had higher residual deformity	0.03	Significant
Age	Presence of residual deformity	No statistically significant correlation	—	Not significant
Foot abduction before tenotomy	Need for additional bone surgery	Greater abduction correlated with not requiring bone surgery	0.007	Significant
Pre-treatment foot flexibility	Treatment results	More rigid feet had worse outcomes	0.02	Significant
AOFAS score	Pre- vs. post-treatment	Significant improvement (32 → 79)	< 0.001	Highly significant
Follow-up duration	AOFAS outcome	Longer follow-up associated with further improvement	—	Descriptive trend
Additional bone surgery	Final outcomes	Need for surgery correlated with worse outcomes; isolated tenotomy had better results	0.002	Significant

Feet with no flexibility in the pre-treatment required significantly more casts (13.0 ± 4.1) than those with mild flexibility (9.5 ± 4.4) or moderate flexibility (3.0 ± 0.0) (p = 0.01).

There was no statistically significant correlation between the number of casts and the variables age and pre-treatment foot rotation (p = 0.54). Patients who required additional bone surgery were older (40.4 ± 11.1 years**)** than those treated only with casts and Achilles tenotomy (29.7 ± 6.7 years**)** (p = 0.04). No significant difference (p = 0.25) between sex and need for additional bone surgery was observed.

The percentage of residual deformity was significantly higher in males (p = 0.03**;** 83.3% > 20.0%; Odds ratio = 20.0; CI 95%, 1.39–301.00).

There was no statistically significant correlation between age and residual deformity.

Feet that did not require additional bone surgery achieved greater forefoot abduction before tenotomy (41.3° ± 5.8°) compared with those that required additional procedures (12.5° ± 11.9°) (p = 0.007).

Pre-treatment foot flexibility was significantly correlated with the results (p = 0.02), with more rigid feet yielding worse outcomes than less rigid feet (moderate flexibility).

The AOFAS scores significantly improved (p < 0.001) from 32.0 **±** 10.0 points (8.0–50.0) to 79.0 **±** 10.0 points (57.0–97.0). Patients with longer follow-up showed improvement in the AOFAS score over time (residual pain, mobility, and gait improved). Complete remission of the callus in the dorsolateral region of the foot was observed, where load was applied before treatment. All patients, including those with residual deformity, were satisfied at the end of treatment (would do it again), and 92% reported no or much less pain than before treatment.

There was a significant association between the need for additional bone surgery and poorer outcomes (p = 0.002). Among surgically treated feet, 90.9% were classified as very good and 9.1% as regular, with no excellent results. In contrast, feet treated with casts and Achilles tenotomy alone achieved 81.8% excellent or very good outcomes, while 4.5% were classified as good and 13.6% as regular.

**Fig 8:**
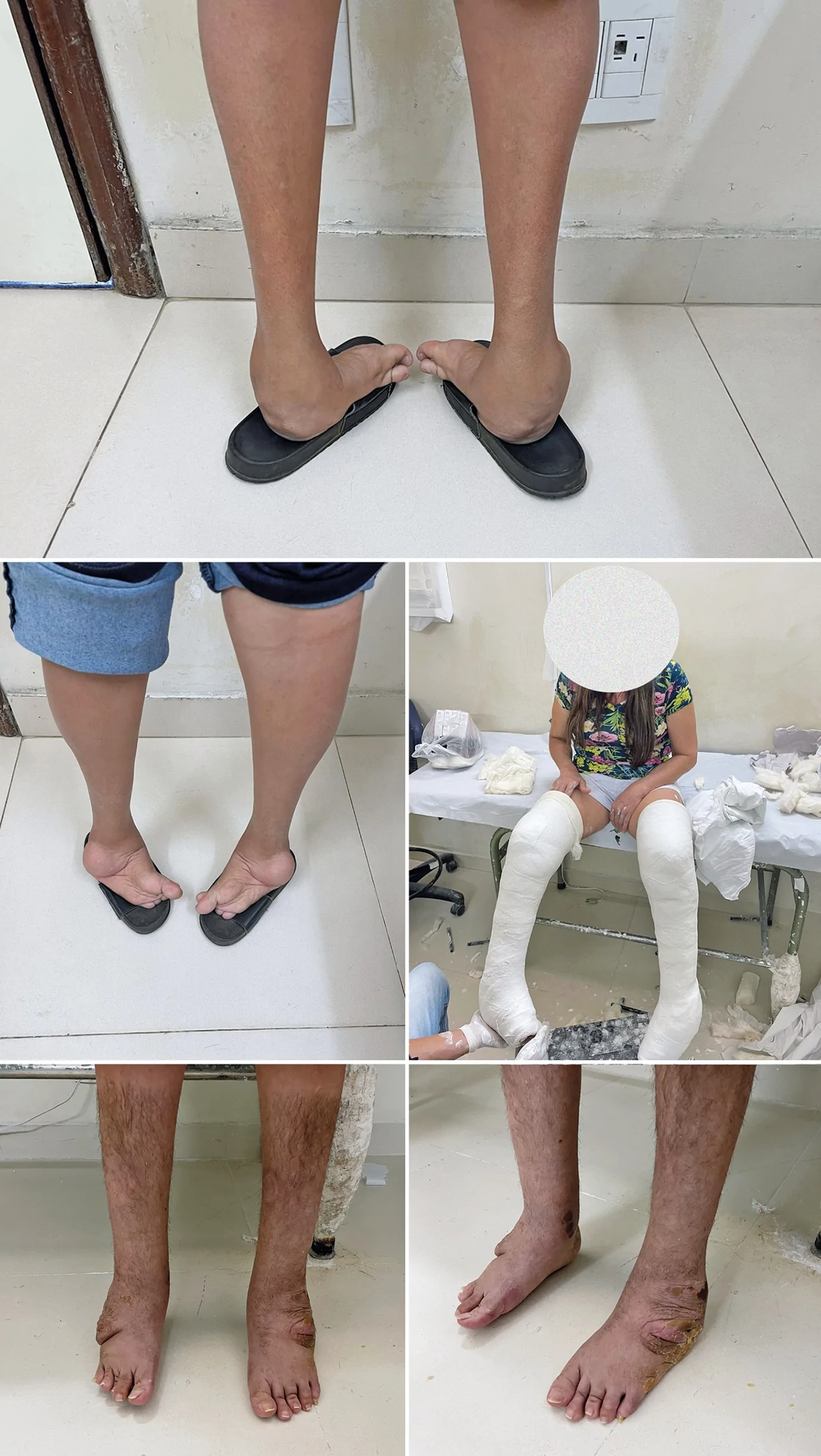
A 53-year-old patient (Patient 21, Table 3). She got a complete correction of her clubfoot with 13 casts and tenotomy (without any bone surgery), being one of our oldest cases treated.

**Fig 9:**
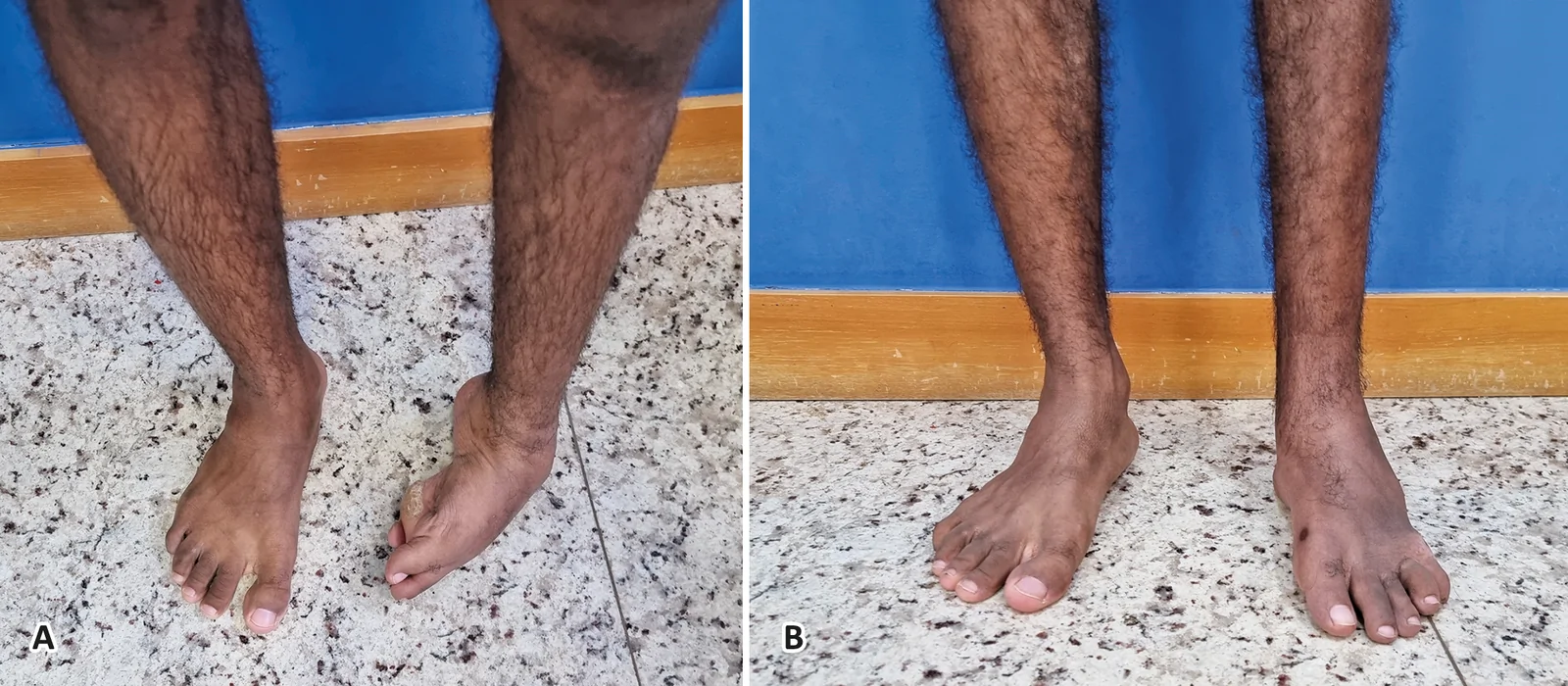
A 28-year-old patient (Patient 8, Table 3) underwent only three plaster casts before Achilles tendon tenotomy, with excellent results and no residual deformities.

**Fig 10:**
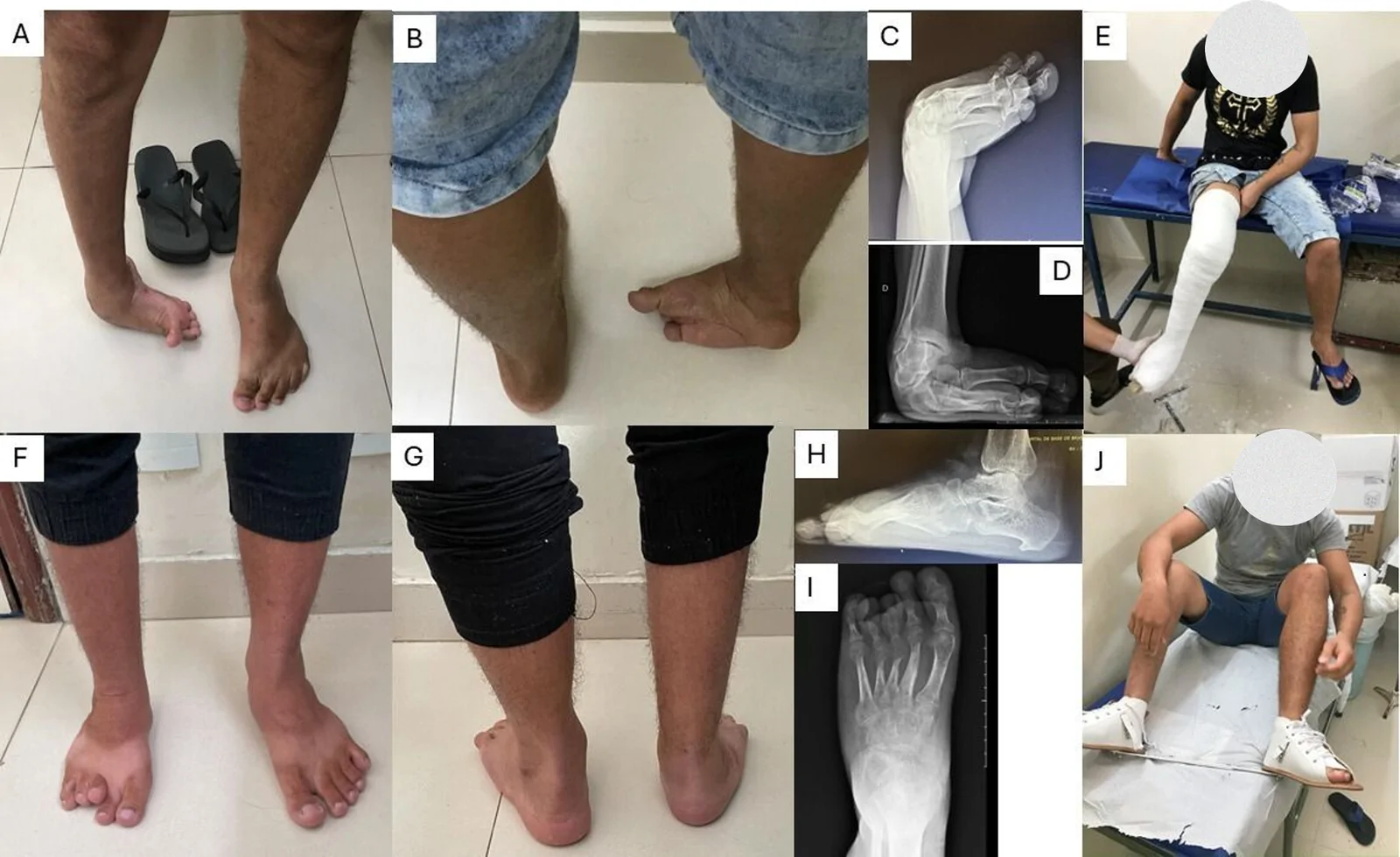
A 22-year-old patient (Patient 16, Table 3). Clinical and radiographic aspects before **(A, B, C, D)** and after treatment **(F, G, H, I)**. Patient underwent 8 casts **(E)** and Achilles tenotomy (without any bone surgery)**.** The patient is using the abduction brace after treatment **(J)**.

Figs 8–10 show adult patients treated only with the Ponseti method (Patient 21–53 years old), (Patient 8–28 years old), and (Patient 16–22 years old), respectively.

## Discussion

The high level of patient satisfaction, the feasibility of using tennis shoes for the first time, and the low rate of complications support continued use of the Ponseti method for neglected clubfoot in adults.

The problem of treating surgically neglected clubfoot in adults in developing countries is the large number of patients and the shortage of specialized surgeons. In addition, talectomy or Ilizarov devices have been reported to lead to residual deformities, stiff and painful feet with cosmetically unacceptable scars [16,20]. The feet operated on unilaterally with the talectomy technique showed a significant discrepancy in lower-limb length [16]. This requires a method that yields better results and is less complex, and that can be readily applied to larger populations [4].

Some of our patients were in their fifties, and our 53-year-old patient is the oldest case reported in the literature to date, treated only with Ponseti casts and Achilles tenotomy. One hypothesis is that the Ponseti method may carry a lower risk of circulatory complications than talectomy or external fixation, although this cannot be confirmed in our small sample. It is not reported in the literature surgically treated patients with neglected clubfoot older than 42 years of age [20].

In our study was observed that pre-treatment foot flexibility was a prognostic factor in adults, influencing outcomes and the number of casts. Our cohort included only one neuromuscular patient, who had a very rigid foot but did not require additional bone surgery and achieved an excellent outcome. The assessment of foot flexibility was based on clinical examination and was therefore inherently subjective. Although it proved useful for stratifying deformity severity and predicting treatment difficulty in this series, its reproducibility and external validity should be evaluated in future studies.

Also, It was found that adult women had fewer residual deformities than men. Another relevant finding is that older cases required more additional bone surgeries; that is, they had less correction capacity with casts than younger cases. In addition, the degree of foot rotation (Fig 3) evaluated before treatment did not show a statistical influence on the number of casts performed, although the patient (number 3 in Table 3) who presented the worst rotation was the one who had more casts, but did not need any associated surgery to the Achilles tenotomy.

In our study, most feet maintained functional mobility and experienced reduced pain compared with pre-treatment status, and 67% of patients avoided major surgery, except for percutaneous Achilles tenotomy. Among the seven patients who required additional bone procedures, prior correction with casts had already achieved partial realignment, reducing the magnitude of subsequent surgery. These procedures were relatively simple—often requiring only Kirschner wire fixation—and contrasted with more technically demanding surgeries such as talectomy or external fixation.

The absence of intra-treatment radiographs limited our ability to determine whether flat-top talus was associated with the need for additional bone surgery.

Residual deformity was present in 14 of 33 feet (42.4%) at the end of follow-up, and most deformities were mild. At the patient level, 10 of 21 patients (47.6%) had residual deformity in at least one foot.

Among our patients, we found that feet with the highest degree of abduction before Achilles tenotomy during the plastering phase had significantly fewer additional bone surgeries. Ponseti reports on the importance of reaching 70° of foot abduction in newborns before tenotomy [6]. Despite this, in our study, some feet with abduction of 15° to 55° before tenotomy did not exhibit residual deformity after equinus correction. Patients who underwent additional bone surgery had worse outcomes than those who underwent only the Ponseti method; however, randomized controlled trials would be ideal to confirm this finding.

In addition, we applied a new angle, the tibiocalcaneal, to assess the degree of the calcaneus equinus, which was easy to measure. Achilles tenotomy was sufficient to correct equinus in all cases (except in one, where an osteotomy was performed in the distal tibia).

Because patients who adhered to the brace protocol experienced fewer recurrences, we recommend the use of a foot abduction brace in adults, similar to standard practice in newborns and children [6,10].

The AOFAS score for neglected clubfoot in adults has limitations. In our opinion, the most important factor is achieving deformity correction, but alignment accounts for only 10% of the reference score. We believe that the AOFAS score should place less emphasis on stability in non-treated adult clubfoot patients, as all of our patients had stable feet before and after treatment. In addition, the AOFAS score improved during follow-up because joint mobility, pain, and tolerance for prolonged walking improved in most cases, even after 12 months.

We also believe that some degree of bone remodeling may occur in adult patients, although to a lesser extent than in children.

Our study has limitations, including a small sample size, the absence of a comparison group treated with alternative surgical techniques, limited long-term follow-up, and the subjective assessment of foot flexibility, recurrence, and residual deformity. Future research should also explore the applicability of this protocol in adults who previously underwent posteromedial release and now present with residual or recurrent deformities.

## Conclusion

The Ponseti method, with additional bone surgery when required for residual deformities, proved to be a viable and effective treatment strategy for neglected clubfoot in adults.

## Supporting information

S1 File Videos legends. Treatment of adult neglected clubfoot with Ponseti Method: consecutive case series of thirty-three feet.
(DOCX)

S1 Video Example of Achilles tenotomy done under spinal anesthesia and sedation in an adult patient. Second session of the video shows the dorsiflexion maneuver just after Achilles tenotomy (same patient).
(MP4)

S2 Video Patient’s 1 (Table 3) functional mobility and standing on tiptoe after treatment. (MP4)

S3 Video Shows patient 16 (Table 3) gait before and after treatment. (MP4)

S4 Video Example of flexibility assessment of a very flexible clubfoot that needed only three casts. Patient 8 (Table 3).
(MP4)

S5 Video Example of very rigid clubfoot associated to rigid claw toes that needed 25 casts and Achilles tenotomy. Patient 3 (Table 3).
(MP4)

S6 Video A 28-year-old patient (Patient 8, Table 3) who underwent only three plaster casts before Achilles tendon tenotomy, with excellent results and no residual deformities. Video shows him walking before treatment, and running and jumping after treatment.
(MP4)

S7 Video 53-year-old patient painful gait before treatment (Patient 21, Table 3). She got a complete correction of her clubfoot with 13 casts and tenotomy (without any bone surgery), being one of our oldest case treated.
(MP4)

S8 Video Shows her callosity in the left foot before treatment. Videos 7–12 show the same patient.
(MP4)

S9 Video Little flexibility of the right foot and moderate on the left. Videos 7–12 show the same patient.
(MP4)

S10 Video Walking cast boot five weeks after Achilles tenotomy. Videos 7–12 show the same patient.
(MP4)

S11 Video Surprise donation of her first tennis shoes. Videos 7–12 show the same patient. (MP4)

S12 Video Shows the 53 year old patient walking barefoot and doing physiotherapy 35 days after the last cast was removed. Videos 7–12 show the same patient.
(MP4)

S1 Dataset Supporting Information (S2 Dataset). (XLSX)
