# Supplementary material for: Treatment of adult neglected clubfoot with Ponseti method: Consecutive case series of thirty-three feet
Source: PLoS One. 2026 Sep 15;21(9):e0358249. doi: 10.1371/journal.pone.0358249 (PMC13577333; doi:10.1371/journal.pone.0358249)
Supplement: Supplementary file 1 [file pone.0358249.s001.docx]

## **Treatment of adult neglected clubfoot with Ponseti Method: consecutive case series of thirty-three feet**

**S1. Videos legends**

**Video 1.** Example of Achilles tenotomy done under spinal anesthesia and sedation in an adult patient. Second session of the video shows the dorsiflexion maneuver just after Achilles tenotomy (same patient).

**Video 2.** Patient’s 1 (Table 3) functional mobility and standing on tiptoe after treatment.

**Video 3.** Shows patient 16 (Table 3) gait before and after treatment.

**Video 4.** Example of flexibility assessment of a very flexible clubfoot that needed only three casts. Patient 8 (Table 3).

**Video 5.** Example of very rigid clubfoot associated to rigid claw toes that needed 25 casts and Achilles tenotomy. Patient 3 (Table 3).

**Video 6**. A 28-year-old patient (Patient 8, Table 3) who underwent only three plaster casts before Achilles tendon tenotomy, with excellent results and no residual deformities. Video shows him walking before treatment, and running and jumping after treatment.

**Video 7**. 53-year-old patient painful gait before treatment (Patient 21, Table 3) . She got a complete correction of her clubfoot with 13 casts and tenotomy (without any bone surgery), being one of our oldest case treated.

**Video 8**. Shows her callosity in the left foot before treatment. Videos 7 to 12 show the same patient.

**Video 9.** Little flexibility of the right foot and moderate on the left. Videos 7 to 12 show the same patient.

**Video 10**. Walking cast boot five weeks after Achilles tenotomy. Videos 7 to 12 show the same patient.

**Video 11.** Surprise donation of her first tennis shoes. Videos 7 to 12 show the same patient.

**Video 12** Shows the 53 year old patient walking barefoot and doing physiotherapy 35 days after the last cast was removed. Videos 7 to 12 show the same patient.
