# Supplementary material for: Treatment of adult neglected clubfoot with Ponseti method: Consecutive case series of thirty-three feet
Source: PLoS One. 2026 Sep 15;21(9):e0358249. doi: 10.1371/journal.pone.0358249 (PMC13577333; doi:10.1371/journal.pone.0358249)
Supplement: Supplementary file 2 [file pone.0358249.s016.docx]

**Review of PONE**

**PONE-D-25-14281
"Treatment of adult neglected clubfoot with Ponseti Method: consecutive case series of thirty-three feet"**

**Congratulations, it is a very important paper and deserves to be published.**

**Below are just a few points that can be improved.**

Discussion

239 - and the “shortage of qualified surgeons” – this is a dangerous sentence. Lots of surgeons do think that patient could loose walking habilities being so much time without walking, and are afraid to do so, but not because of expertise…

251 – “In our study, for the first time,” – this repeating for the first time is not academically appropriated. This is to be judged by the readers. Please take out these so repeated and inadequate terms.

289 – “stability in neglected clubfoot patients” – It would be nice to take out the neglected term, considered pejorative and negative – better to substitute for non treated adults

It would be nice to have a point in the discussion about bony remodeling, based in what you have seen in the clinical and radiographic aspects of your patients. This is to make a correspondence between Pirani´s 2001 MRI article, this

**Nogueira MP**, Amaral DT. How much remodelling is possible in a clubfoot treatment? Magnetic resonance imaging study in a 7-year-old child. J Limb Lengthen Reconstr. 2018;4(1):49-54.

And yours.
