# Supplementary material for: Treatment of adult neglected clubfoot with Ponseti method: Consecutive case series of thirty-three feet
Source: PLoS One. 2026 Sep 15;21(9):e0358249. doi: 10.1371/journal.pone.0358249 (PMC13577333; doi:10.1371/journal.pone.0358249)
Supplement: Supplementary file 3 [file pone.0358249.s017.docx]

Dear Editor-in-Chief,

## Thank you for considering the paper "**Treatment of adult neglected clubfoot with Ponseti Method: consecutive case series of thirty-three feet**", for publication in the *PLos One* and for allowing us to re-submit this revised manuscript.

The authors are grateful to the reviewers for the careful appraisal, positive comments, and helpful criticisms. Suggested changes have been addressed. We believe that the quality of the manuscript has been improved and hope that it now meets the quality required for publication in the *PLos One*.

A detailed point-by-point response is given below.

We are looking forward to your decision regarding the suitability of the revised version of this paper for the Journal.

Thank you very much in advance,

Journal Requirements:

When submitting your revision, we need you to address these additional requirements.

1. Please ensure that your manuscript meets PLOS ONE's style requirements, including those for file naming. The PLOS ONE style templates can be found at

https://journals.plos.org/plosone/s/file?id=wjVg/PLOSOne_formatting_sample_main_body.pdf and

<https://journals.plos.org/plosone/s/file?id=ba62/PLOSOne_formatting_sample_title_authors_affiliations.pdf>

**Response:** We have reviewed the PLOS ONE formatting guidelines and revised the manuscript and file names accordingly to ensure full compliance with the journal’s style requirements.

2. We noted in your submission details that a portion of your manuscript may have been presented or published elsewhere. “https://ssrn.com/abstract=4986324 or http://dx.doi.org/10.2139/ssrn.4986324” Please clarify whether this publication was peer-reviewed and formally published. If this work was previously peer-reviewed and published, in the cover letter please provide the reason that this work does not constitute dual publication and should be included in the current manuscript.

**Response:** The SSRN document (DOI: 10.2139/ssrn.4986324) is a non–peer-reviewed preprint posted solely to facilitate early academic dissemination. It has not undergone journal editorial review, peer review, or formal publication. The current submission to PLOS ONE represents the first peer-reviewed version of this work. Therefore, this manuscript does not constitute dual publication.

3. Your ethics statement should only appear in the Methods section of your manuscript. If your ethics statement is written in any section besides the Methods, please move it to the Methods section and delete it from any other section. Please ensure that your ethics statement is included in your manuscript, as the ethics statement entered into the online submission form will not be published alongside your manuscript.

**Response:** Thank you for the clarification. The ethics statement, mentioned elsewhere in the manuscript, has been removed and is now included exclusively in the Methods section (lines 72-73) as follows: “All patients included in this manuscript have given written informed consent (as outlined in PLOS consent form) to publish these case details, including the images in Figures and Videos (S1 Videos).”

4. We note that Figure 1 - 8 includes an Image of a patient in the study. 
As per the PLOS ONE policy (http://journals.plos.org/plosone/s/submission-guidelines#loc-human-subjects-research) on papers that include identifying, or potentially identifying, information, the individual(s) or parent(s)/guardian(s) must be informed of the terms of the PLOS open-access (CC-BY) license and provide specific permission for publication of these details under the terms of this license. Please download the Consent Form for Publication in a PLOS Journal (http://journals.plos.org/plosone/s/file?id=8ce6/plos-consent-form-english.pdf). The signed consent form should not be submitted with the manuscript, but should be securely filed in the individual's case notes. Please amend the methods section and ethics statement of the manuscript to explicitly state that the patient/participant has provided consent for publication: “The individual in this manuscript has given written informed consent (as outlined in PLOS consent form) to publish these case details”.

If you are unable to obtain consent from the subject of the photograph, you will need to remove the figure and any other textual identifying information or case descriptions for this individual.

**Response:** Thank you for this clarification. The manuscript has been updated to explicitly state that the individual in the figures provided written informed consent for publication under the terms of the PLOS open-access (CC-BY) license. This statement now appears in the Methods section (lines 72-73). No images were removed, as the required consent was obtained and securely filed as recommended.

5. If the reviewer comments include a recommendation to cite specific previously published works, please review and evaluate these publications to determine whether they are relevant and should be cited. There is no requirement to cite these works unless the editor has indicated otherwise.

**Response**: Thank you for your comment. The reviewers did not request the citation of any specific previously published work. Nevertheless, we re-examined the recent medical literature to ensure that no relevant studies on the treatment of adult neglected clubfoot published in the past five years were overlooked.

Additional Editor Comments:

Experts in this field have reviewed your article. There have been some concerns regarding the method and results of your study outlined by the reviewers. Please answer them carefully, in a point-by-point manner. Moreover, your article seems to require English editing services.

**Response**: Thank you for your comment. The manuscript has undergone a thorough English language revision. All major addition phrases and/or paragraphs were highlighted in yellow in the revised version.

[Note: HTML markup is below. Please do not edit.]

Reviewers' comments:

Reviewer's Responses to Questions

**Comments to the Author**

1. Is the manuscript technically sound, and do the data support the conclusions?

The manuscript must describe a technically sound piece of scientific research with data that supports the conclusions. Experiments must have been conducted rigorously, with appropriate controls, replication, and sample sizes. The conclusions must be drawn appropriately based on the data presented.

Reviewer #1: Yes

Reviewer #2: Partly

Reviewer #3: Yes

2. Has the statistical analysis been performed appropriately and rigorously?

Reviewer #1: I Don't Know

Reviewer #2: I Don't Know

Reviewer #3: Yes

3. Have the authors made all data underlying the findings in their manuscript fully available?

The [PLOS Data policy](http://www.plosone.org/static/policies.action#sharing) requires authors to make all data underlying the findings described in their manuscript fully available without restriction, with rare exception (please refer to the Data Availability Statement in the manuscript PDF file). The data should be provided as part of the manuscript or its supporting information, or deposited to a public repository. For example, in addition to summary statistics, the data points behind means, medians and variance measures should be available. If there are restrictions on publicly sharing data—e.g. participant privacy or use of data from a third party—those must be specified.

Reviewer #1: Yes

Reviewer #2: Yes

Reviewer #3: Yes

4. Is the manuscript presented in an intelligible fashion and written in standard English?

PLOS ONE does not copyedit accepted manuscripts, so the language in submitted articles must be clear, correct, and unambiguous. Any typographical or grammatical errors should be corrected at revision, so please note any specific errors here.

Reviewer #1: Yes

Reviewer #2: No

Reviewer #3: Yes

5. Review Comments to the Author

Please use the space provided to explain your answers to the questions above. You may also include additional comments for the author, including concerns about dual publication, research ethics, or publication ethics. (Please upload your review as an attachment if it exceeds 20,000 characters)

Reviewer #1: Dear Editor,
Thank you for the opportunity to review this manuscript. The authors present an important and timely study on a challenging clinical problem. The use of the Ponseti method for neglected clubfoot in an adult population is a valuable area of research, and this case series is a significant contribution. The manuscript is well-written, but I have several comments and questions that the authors should address to strengthen their work.
My review is organized by section below.
Introduction
The introduction effectively establishes the clinical problem. The literature review is logical and concise, providing a strong justification for the study, and the objectives are clearly stated.
• Minor Suggestion: The authors use the term "medium number of casts." I would recommend replacing this with the more standard scientific terminology of "mean" or "average number of casts."

**Response:** Thank you for this suggestion. The manuscript has been revised for clarity and adherence to standard scientific terminology. The term “medium number of casts” was replaced with “mean number of casts,” as noted in line 58.

________________________________________
Methods
This section raises several important questions regarding patient selection and methodological clarity.
• Exclusion Criteria: The rationale for excluding patients with a history of thrombosis requires clarification. Could these patients not be managed with prophylactic anticoagulation, similar to the protocol used for other high-risk patients in the study?

**Response:** Thank you for your comment. Although patients with a history of thrombosis could theoretically have been managed with prophylactic anticoagulation, the attending surgeon (main author) considered a prolonged casting period a major risk factor and therefore excluded such patients for safety reasons. This decision aimed to avoid exposing individuals with a documented thrombophilic history to an elevated risk of recurrent thromboembolic events. We have clarified this rationale in the revised Methods section (lines 75-81): “Five patients were excluded due to conditions associated with a substantially increased risk of thrombosis (previous personal thrombosis or significant varicose veins, n = 2) or arterial disease (diabetes mellitus or chronic smoking, n = 3). Although such patients could theoretically be managed with prophylactic anticoagulation, the treating surgeon considered these risk factors incompatible with the prolonged casting period required by this protocol and therefore excluded them for safety reasons.”

• Anticoagulation Guidelines: The manuscript should cite the specific guidelines or criteria used for prescribing anticoagulants to patients who were overweight or over 50 years old.

**Response:** Thank you for your comment. Anticoagulation for overweight patients and those over 50 years of age was prescribed after consultation with the vascular specialist (angiologist) at our institution. In these cases, prophylaxis was considered a relative indication and based on an individualized risk assessment rather than a formal guideline-directed protocol.

This approach is consistent with the findings of the TILLIRI Study (Halloran et al., 2025), which emphasized that prophylaxis decisions during lower-limb immobilization are often guided by individualized risk stratification rather than routine anticoagulation. Obesity is a well-established risk factor for venous thrombosis, and although age is sometimes debated in risk-stratification models, Silverstein et al. demonstrated a clear increase in venous thromboembolism incidence beginning after age 45 and rising progressively thereafter.

**Response:** Thank you for your comment. We have clarified this rationale in the revised Methods section (lines 107-109): “In these cases, anticoagulation was considered a relative indication and prescribed based on individualized risk assessment. ”

• Flexibility Assessment: The assessment of pre-treatment flexibility is described qualitatively ('little', 'moderate', 'rigid'). The authors should discuss why a quantitative classification system, such as the DeMilio classification for clubfoot, was not used to provide a more objective and reproducible measure.

**Response:** Thank you for your comment. We believe the reviewer is referring to the Diméglio classification, which is widely used in infants with congenital clubfoot. However, this system was not appropriate for our study population for several reasons. First, the Diméglio score does not quantify foot flexibility, which was one of the specific parameters assessed in our adults with long-standing, untreated deformities. Second, this classification has been shown to exhibit substantial intra- and inter-observer variability and does not reliably predict prognosis in neglected or adult cases. To our knowledge, no existing classification specifically evaluates pre-treatment flexibility in adult or inveterate clubfoot. For this reason, we developed a simplified flexibility grading system tailored to this population's characteristics. In addition, we quantified the degree of foot rotation relative to the leg and applied the AOFAS score to complement the clinical evaluation. We have clarified this rationale in the revised Results section (lines 203-204): “Feet with no flexibility in the pre-treatment required significantly more casts (13.0 ± 4.1) than those with mild flexibility (9.5 ± 4.4) or moderate flexibility (3.0 ± 0.0) (p = 0.01).”

• Neuromuscular Conditions: The manuscript needs to be more explicit about the presence of associated neuromuscular conditions. While one case of myelomeningocele is mentioned, it is unclear if other conditions like arthrogryposis were present in the cohort or were part of the exclusion criteria.

**Response:** Thank you for your comment. Neuromuscular conditions, such as arthrogryposis or myelomeningocele, were not among the exclusion criteria. This was a consecutive adult cohort, and only one patient with a neuromuscular condition (myelomeningocele) presented for treatment during the study period. We have clarified this rationale in the revised Discussion section (lines 251-253): “Our cohort included only one neuromuscular patient, who had a very rigid foot but did not require additional bone surgery and achieved an excellent outcome.”

• Radiographic Analysis: The pre-operative radiographic analysis did not include an assessment for flat-top talus. Given that this finding can probably predict the need for more extensive surgery beyond an Achilles tenotomy, its presence or absence is valuable information that should be reported and discussed.

**Response:** Thank you for your comment. Pre-treatment radiographs were obtained at baseline and after completion of the treatment protocol; however, intra-treatment radiographs taken immediately before the Achilles tenotomy or before any additional surgical procedure were not included in our routine protocol. Therefore, a systematic assessment of flat-top talus at the specific surgical decision point was not feasible. Moreover, although a flat-top talus has been described in pediatric populations as a possible indicator of long-standing deformity, the evidence supporting its prognostic value in adults with neglected or inveterate clubfoot remains limited. In our cohort, pre-treatment radiographs did not show a consistent association between the presence or absence of a flattened talar dome and the eventual need for more extensive bone surgery beyond an Achilles tenotomy. We have now clarified this point in the revised manuscript and have added this issue as a methodological limitation in the Discussion section (lines 267-268): “The absence of intra-treatment radiographs limited our ability to determine whether flat-top talus was associated with the need for additional bone surgery.”

________________________________________
Results
The presentation of the results could be enhanced with more quantitative detail.
• Defining Outcomes: The terms "relapse" and "residual deformity" lack quantitative definition. The authors should specify how these were measured or graded. Was there a standardized method to quantify the degree of remaining or recurrent deformity?

**Response:** Thank you for this important observation. We did not quantitatively measure or grade relapse or residual deformity. These outcomes were determined based on clinical assessment. We acknowledge this is a study limitation, as the evaluation of residual deformity and relapse was subjective and lacked a standardized grading method. This limitation has now been explicitly addressed in the revised Discussion section (lines 291-293): “Our study has limitations, including a small sample size, the absence of a comparison group treated with alternative surgical techniques, limited long-term follow-up, and subjective assessment of recurrence and residual deformity.”

• Surgical Decision-Making: The decision-making process for performing additional surgery after casting and tenotomy is unclear. The results table presents an apparent contradiction where some patients with 'mild' residual deformity underwent surgery, while another with a 'moderate' deformity (Patient 11) did not. The criteria for this crucial clinical decision must be explicitly detailed.

**Response:** Thank you for highlighting this important point. The decision-making process for performing additional surgery after casting and tenotomy is described in the Methods section (lines 128-131). To enhance clarity, we have prepared a flowchart summarizing the decision pathway, which was included as Fig 5. “The surgical procedure was followed by a long-leg cast for three weeks. After this period, a short cast was instrumented with weight-bearing for three to six weeks, and, finally, the double abduction orthosis, with the feet in 40° of external rotation, was worn for eight hours/day for one year (Fig 5).”

Regarding the presentation of outcomes, residual deformity was assessed at the last follow-up visit after completion of treatment (casts and tenotomy, or casts, tenotomy, and additional surgery), as shown in the column “Residual deformity at last follow-up” in Table 3. It is important to note that patients may present with residual deformity after casting and Achilles tenotomy alone, while some patients who underwent additional surgery may show no residual deformity at follow-up.

Specifically for Patient 11, the information in the “Partial recurrence/brace protocol adherence” column of Table 3 clarifies the apparent discrepancy. This patient experienced a partial recurrence, resulting in a moderate residual deformity at the last follow-up, despite no residual deformity immediately after the final cast. This finding is relevant, as it represents, to our knowledge, the first report demonstrating that relapses may also occur in adults treated for neglected or inveterate clubfoot.

________________________________________
Discussion
The discussion is well-grounded but would benefit from addressing the study's limitations more directly.
• Impact of Sample Size: The discussion would be strengthened by a more thorough examination of the study's limitations, particularly the small sample size and its potential impact on the statistical power and generalizability of the findings. While acknowledging the difficulty of recruiting such a cohort is fair, the implications of this limitation must be addressed.

• Lack of a Comparative Group: The authors should also discuss the lack of a comparative group treated with standard surgical methods, which is a key limitation when evaluating the efficacy of the proposed treatment.

**Response:** Thank you for this insightful comment. We have expanded our discussion of the study’s limitations, particularly regarding the small sample size and its implications for statistical power and generalizability. Although assembling a cohort of adults with untreated congenital clubfoot is inherently challenging, we agree that the limited sample reduces the strength of the conclusions and may not fully represent the broader population of patients with neglected or inveterate deformities. We have also emphasized the absence of a comparison group treated with standard surgical methods, which restricts our ability to directly evaluate the relative efficacy of the proposed approach. This limitation has now been explicitly addressed in the revised Discussion section (lines 291-293): “Our study has limitations, including a small sample size, the absence of a comparison group treated with alternative surgical techniques, limited long-term follow-up, and subjective assessment of recurrence and residual deformity.”

________________________________________
Suggestion for Future Research
The authors have laid the groundwork for a valuable future study. Their current cohort consists of previously untreated patients. However, many patients in this age group may have undergone procedures like posteromedial release in the past and now present with residual or recurrent deformities. Since this study demonstrates the feasibility of the Ponseti method even in rigid feet, applying a similar protocol to this post-surgical population would be a logical and impactful next step.

**Response:** Thank you for this excellent suggestion. We agree that applying a similar casting protocol to adults with residual or recurrent deformities after prior posteromedial release is an important area for future research. In our clinical practice, we have treated a small number of such patients, but the process is considerably more challenging due to markedly reduced soft-tissue flexibility and heightened concern for circulatory compromise from scarring in the posteromedial ankle region. In addition, we do not initiate casting when there is evidence of bony synostosis or prior arthrodesis, as these conditions substantially limit the potential for correction. We have now incorporated this point into the revised Discussion to highlight the relevance and feasibility of investigating this post-surgical population in future studies (lines 293-295): “Future research should also explore the applicability of this protocol in adults who previously underwent posteromedial release and now present with residual or recurrent deformities.”

In summary, this is a noteworthy paper that, with revision, will be a strong addition to the literature. I look forward to seeing a revised version that addresses these points.
 **Response:** Thank you for your consideration and comments.

Reviewer #2: Dear Authors,

Your manuscript provides valuable insights into the treatment of neglected clubfoot in adults. However, I believe the paper requires major revisions as outlined below:

The manuscript contains several instances of language use that are not suitable for a scientific paper. I recommend seeking assistance from a native English speaker or using AI tools only for grammar checking and paraphrasing.

**Response**: Thank you for your comment. The manuscript has undergone a thorough English language revision. All major addition phrases and/or paragraphs were highlighted in yellow in the revised version.

The introduction does not follow a standard academic structure and lacks logical flow. For instance, the content in lines 64–65 would fit better in the first paragraph, while lines 57–63 could be used to describe current treatment methods for neglected clubfoot in adults. I strongly suggest reorganizing the introduction into 3–4 coherent paragraphs following the conventional structure (background, problem statement, existing knowledge, and study aim).

**Response:** Thank you for your comment. The content from lines 64-68 is now placed in the Introduction's first paragraph (lines 39-43).

Lines 141–142: The sentence “All patients received a pair of sneakers donated by the main author, as the first sneaker in life after the removal of the last cast” is not appropriate and ethical for inclusion in a scientific paper and should be removed.

**Response:** Thank you for your comment. The sentence was removed.

The results section reports numerous p-values but lacks corresponding means and standard deviations. Please include these descriptive statistics where applicable.

**Response:** Thank you for your comment. a) The following sentence: “There was a significantly greater need for cast changes in the feet with less flexibility in the pretreatment (p=0.01).” was changed by this one in lines 203-204: “Feet with no flexibility in the pre-treatment required significantly more casts (13.0 ± 4.1) than those with mild flexibility (9.5 ± 4.4) or moderate flexibility (3.0 ± 0.0) (p = 0.01).”; b) the following sentence: “There was a significant difference between age and the need for additional bone surgery, with higher values for age with a positive correlation with the need for additional surgery (p = 0.04).” was changed by this one in lines 206-208: “Patients who required additional bone surgery were older (**40.4 ± 11.1 years**) than those treated only with casting and Achilles tenotomy **(29.7 ± 6.7 years)** (p = 0.04).”; c) In lines 210-211 is now written: “The percentage of residual deformity was significantly higher in males (p = 0.03**;** 83.3% > 20.0%; Odds ratio = 20.0; IC 95%, 1;39-301.00).”; d) The following sentence: “For higher values of foot abduction after the cast period and before Achilles tendon tenotomy, there was a statistically significant correlation (p=0.007) with not needing to perform additional bone surgeries.” was changed to this on in lines 213-215: “Feet that did not require additional bone surgery achieved greater forefoot abduction before tenotomy (**41.3° ± 5.8°**) compared with those that required additional procedures (**12.5° ± 11.9°**) (p = 0.007).”; e) See lines 218-219: “The AOFAS scores significantly improved (p < 0.001) from 32.0 ± 10.0 points (8.0-50.0) to 79.0 ± 10.0 points (57.0-97.0).”; f) The following sentence: “There was a significant correlation between the need for additional bone surgery and worse results, while those who only underwent tenotomy of the Achilles tendon had better results (p=0.002).” was changed to in lines 225-229: “There was a significant association between the need for additional bone surgery and poorer outcomes (p = 0.002). Among surgically treated feet, 45.4% were classified as very good and 9.1% as regular, with no excellent results. In contrast, feet treated with casts and Achilles tenotomy alone achieved 81.8% excellent or very good outcomes, while 4.5% were classified as good and 13.6% as regular.”

Line 175: The term “Medium” is not standard in statistical reporting and should be replaced with the correct term (e.g., “median” or “mean,” depending on the intended meaning).

**Response:** Thank you for your comment. It was changed to mean.

The discussion section primarily reiterates the authors’ findings without adequately comparing them to existing literature or alternative treatment methods. Please revise this section to include a critical comparison with relevant studies.

**Response:** Thank you for this important observation. We agree that a critical comparison with the existing literature is essential; however, our ability to perform such an analysis is fundamentally limited by the lack of published studies evaluating the use of the Ponseti method in adults with neglected congenital clubfoot. To our knowledge, no previous reports describe the outcomes of applying this method in a population comparable in age, deformity severity, or chronicity to our cohort.

The few available studies that describe alternative treatment strategies—such as Ilizarov external fixation, talectomy, or other extensive surgical procedures—are not directly comparable, as they involve substantially younger adults and smaller retrospective series. We conducted an additional literature search during revision and were unable to identify new studies that provide a meaningful basis for direct comparison.

Some of the included patient images show faces that are partially identifiable. This is unprofessional and raises ethical concerns. All identifiable features must be removed or blurred in accordance with ethical publishing standards.

**Response:** Thank you for this important observation. All identifying facial features in patient figures have been blurred, and a new statement has been added to the Methods section to ensure compliance with the PLOS ONE consent policy.

Reviewer #3: Dear authors, thank you for your manuscript. Some concerns and suggestions for improvement are in order, and I have laid them out line by line:

Introduction
Line 58: Please include a few sentences explaining the Ponseti technique, particularly emphasizing its principles and rationale.

**Response:** Thank you for your comment. We have added in the revised Introduction section (lines 46-47): “It is based on serial casting, followed by Achilles tenotomy and bracing to prevent recurrences [3-7].”

Method
1. Please specify the study design. Was it a prospective cohort study or another type?

**Response:** Thank you for your comment. We have added in the revised Methods section (lines 69-70): “This retrospective, consecutive case series was conducted at a tertiary hospital from January 2019 to August 2024, with medical records analyzed.”

2. Including a flowchart illustrating the patient selection and inclusion process (from 43 initial patients to the final 21) would enhance clarity. If this is not feasible, please elaborate more. Take Line 100 for instance, six patients were “waiting for treatment”, it is unclear what they were waiting for and why they were excluded.

**Response:** Thank you for this helpful suggestion. We have now created a flowchart illustrating the patient selection and inclusion process, from the initial 43 patients to the final cohort of 21. This has been added as Fig 1 in the revised Methods section (lines 89-90), providing a clearer visualization of how patients were assessed and included: “The selection process is shown in Fig 1.**”**

3. Some grammatical corrections are needed. Specifically lines 121, 183, and 249-251.

**Response**: Thank you for your comment. The manuscript has undergone a thorough English language revision. All major addition phrases and/or paragraphs were highlighted in yellow in the revised version.

4. Line 125: The manuscript states that up to 15 cast changes were performed. Considering that each cast was applied for seven days, this equates to approximately 3.5 months of serial casting which raises concerns of knee stiffness due to prolonged immobilization. Please clarify.

**Response:** Thank you for raising this important point. We agree that prolonged casting may raise concerns regarding knee stiffness. In our cohort, some stiffness was indeed observed immediately after cast removal; however, all patients regained their pre-treatment range of motion within a few weeks without requiring specific interventions. This clarification has been added to the revised manuscript (lines 186-188): “Although some stiffness and discomfort were reported in the knees immediately after the last cast, all patients regained their pre-treatment range of motion and reported no pain at the last follow-up.”

5. Line 130: The inclusion of a patient with myelomeningocele is questionable. Managing clubfoot in such patients is challenging, often requiring multiple tendon transfers or osteotomies to achieve a plantigrade foot. Serial casting in an adult with myelomeningocele is unlikely to be effective.

**Response:** Thank you for raising this point. We acknowledge that managing clubfoot in patients with myelomeningocele is typically challenging and often requires multiple soft-tissue or bone procedures to achieve a plantigrade foot. However, in our cohort, the included patient with myelomeningocele responded unexpectedly well to the casting protocol. As illustrated in Fig 6, correction was achieved with casting and Achilles tenotomy alone, and the final clinical assessment showed mild overcorrection. This case suggests that, although uncommon, certain adults with neuromuscular conditions may still benefit from a Ponseti-based approach.

6. Line 135: Please clarify the criteria used to determine when patients required surgical intervention. How many cast changes were performed before surgery was indicated?

**Response:** Thank you for this important question. As stated in the revised Methods section (lines 115-117), a maximum of 15 cast changes was accepted before considering additional procedures. The number of casts applied to each patient is reported in Table 3 (column “Casts number²”). Furthermore, the decision-making process for performing surgery after casting and tenotomy is detailed in lines 146-149. To enhance clarity, we have prepared a flowchart summarizing the decision pathway, which was included as Fig 5.

7. Several patients reportedly had bilateral deformities. What was your protocol for them? Were casts applied simultaneously to both feet, or sequentially?

**Response**: Thank you for your comment. To enhance clarity, one sentence was added in lines 112-113: “Bilateral cases were treated simultaneously.”

8. While the postoperative protocol is described, there is no mention of the post-casting protocol. Please include details such as the need for bracing, type of brace, and duration or schedule of use.

**Response**: Thank you for your comment. That is explained in lines 129-131.

Results
1. Line 175: You mention a median of 11.6 cast changes (range 3–25) before Achilles tenotomy, yet earlier you stated a maximum of 15 cast changes. Please clarify this discrepancy.

**Response**: Thank you for your comment. To enhance clarity, one sentence was added in lines 115-117: “To avoid excessive muscle and bone weakness, the maximum number of cast changes accepted was 15 (except in one patient who asked to continue cast changes).”

2. Lines 175, 185: Replace the term “grade” with “level” throughout for consistency.

**Response**: Thank you for your comment. The term “level” is now replaced with “grade” throughout the manuscript.

3. Line 174, Table 3: Replace the term “little” with “mild” for better consistency.

**Response**: Thank you for your comment. The term “little” is now replaced with “mild” throughout the manuscript.

4. Line 174, Table 3: Define the categories of mild, moderate, and rigid flexibility in the Methods section.

**Response**: Thank you for your comment. The categories were defined throughout the manuscript.

5. Line 200, Table 3: Clarify how you defined mild, moderate, and severe deformities (equinus, cavus, adduction). This should also be described in the Methods section.

**Response:** Thank you for your comment. We may have misunderstood the reviewer’s concern, as line 200 does not reference the severity of deformities. In addition, we did not use quantitative criteria to classify residual or relapsed deformities as mild, moderate, or severe. These assessments were based solely on clinical judgment rather than on standardized grading systems. We recognize that this subjective evaluation represents a methodological limitation, and this limitation has now been explicitly addressed in the revised Discussion section (lines 291-294): “Our study has limitations, including a small sample size, the absence of a comparison group treated with alternative surgical techniques, limited long-term follow-up, and subjective assessment of recurrence and residual deformity.”

6. Lines 210–234: For clarity and better visualization, present the correlation findings in a table format.

**Response:** Thank you for your comment. The text between lines 203 and 229 can be summarized in Table 4. Table 4 is referred to in the text in lines 201-202.

7. The Results mention complications, but there is no information on possible knee joint issues after long-term casting. Please clarify whether any stiffness, pain, or limitation occurred in the knees after cast removal, given the prolonged semi-flexed position during treatment. If no data available, please mention this in the limitations parts.

**Response:** Thank you for raising this important point. We agree that prolonged casting may raise concerns regarding knee stiffness. In our cohort, some stiffness was indeed observed immediately after cast removal; however, all patients regained their pre-treatment range of motion within a few weeks without requiring specific interventions. This clarification has been added to the revised manuscript (lines 186-188): “Although some stiffness and discomfort were reported in the knees immediately after the last cast, all patients regained their pre-treatment range of motion and reported no pain at the last follow-up.”

Discussion
1. Line 239: Begin the discussion by clearly emphasizing the key findings of your study.

**Response:** Thank you for your comment. We moved a paragraph that was at the end of the discussion to its beginning in lines 234-236: “The high level of patient satisfaction, the feasibility of using tennis shoes for the first time, and the low rate of complications support continued use of the Ponseti method for neglected clubfoot in adults.”

2. Lines 247–251: The Ponseti method can be technically challenging even in neonates due to the risk of circulatory or skin complications. Numerous reports highlight these risks. Therefore, the claim that “the Ponseti method probably has fewer circulatory complications than talectomy or external fixator devices and can be used in older populations” is not sufficiently supported given the limited number of cases and potential selection bias.

**Response:** Thank you for this important comment. We agree that our original statement was too strong, given the limited number of cases and the potential for selection bias. We have revised the paragraph to present this point more cautiously. The manuscript now states (lines 245-248): “One hypothesis is that the Ponseti method may carry a lower risk of circulatory complications than talectomy or external fixation, although this cannot be confirmed in our small sample.”

3. Lines 263–269: Casting may not replace surgery for neglected clubfoot; however, it may reduce the magnitude of surgery required. Discuss this potential benefit in your Discussion. You might also compare complication rates and technical demands of complex surgical procedures versus your approach.

**Response**: Thank you for this valuable suggestion. We have expanded the Discussion to address the potential benefit of reducing the magnitude of surgery after casting. As now stated in lines 260-266, the paragraph has been rewritten as follows: “In our study, most feet maintained functional mobility and experienced reduced pain compared with pre-treatment status, and 67% of patients avoided major surgery, except for percutaneous Achilles tenotomy. Among the seven patients who required additional bone procedures, prior correction with casts had already achieved partial realignment, reducing the magnitude of subsequent surgery. These procedures were relatively simple—often requiring only Kirschner wire fixation—and contrasted with more technically demanding surgeries such as talectomy or external fixation.”

The following sentence was redundant and has been removed from the manuscript: *“With the Ponseti method, there was a drastic reduction in the need for surgical procedures, except for Achilles tendon tenotomy.”*

Additionally, as shown in Table 3 (column “Associated bone surgery to Achilles tenotomy”), 67% of patients did not require any bone surgery following Ponseti treatment (casting and tenotomy).

4. Lines 275–276: The statement implying that casting yields better outcomes than surgery could be misleading due to selection bias. Such a claim would require further studies, ideally randomized controlled trials (RCTs).

**Response:** Thank you for this important comment. As noted in the Results, we reported that patients who required additional bone surgery had worse outcomes than those who underwent only casts and percutaneous Achilles tenotomy (lines 206-208). To address the reviewer’s concern, we have revised the Discussion to acknowledge that this finding cannot establish superiority of one approach over the other, and that randomized controlled trials would be needed to confirm such differences (lines 277-278): “…however, randomized controlled trials would be ideal to confirm this finding.”

5. Line 281: Details regarding the brace type, duration, and use should be moved to the Methods section.

**Response:** Thank you for your comment. The technical details regarding brace type, duration, and use have now been moved from the Discussion to the Methods section, as requested. In the Discussion (lines 282-284), we now include only a clinical interpretation of this finding: “Because patients who adhered to the brace protocol experienced fewer recurrences, we recommend the use of a foot abduction brace in adults, similar to standard practice in newborns and children [6,10].”

6. Line 294: Include a dedicated section outlining the limitations of your study. The bare minimum is to mention the small sample size, selection bias, and lack of long-term follow-up. Add suggestions for future research.

**Response:** Thank you for your comment. We have included a dedicated limitations section. As stated in the revised manuscript (lines 291-293): “Our study has limitations, including a small sample size, the absence of a comparison group treated with alternative surgical techniques, limited long-term follow-up, and subjective assessment of recurrence and residual deformity.” In addition, we have added a brief statement outlining directions for future research, as suggested by the reviewer (lines 293-295): “Future research should also explore the applicability of this protocol in adults who previously underwent posteromedial release and now present with residual or recurrent deformities.”

References
Many of the cited studies are outdated. Please update the reference list with more recent and relevant publications.

**Response:** Thank you for your comment. We conducted an additional literature search to identify more recent studies. However, aside from the references already included in the manuscript, we were unable to find any publications from the last five years that address the treatment of adult neglected clubfoot without prior treatment. To our knowledge, no new evidence has been published on this specific topic.

Overall, this study presents an interesting and innovative adaptation of the Ponseti method. With comprehensive revisions to methodology, data presentation, and language, it could make a valuable contribution to the literature.

**Response:** Thank you for your comment.

6. PLOS authors have the option to publish the peer review history of their article ([what does this mean?](https://journals.plos.org/plosone/s/editorial-and-peer-review-process#loc-peer-review-history)). If published, this will include your full peer review and any attached files.


If you choose “no”, your identity will remain anonymous but your review may still be made public.


**Do you want your identity to be public for this peer review?** For information about this choice, including consent withdrawal, please see our [Privacy Policy](https://www.plos.org/privacy-policy).

Reviewer #1: No

Reviewer #2: **Yes:** Reza Niakan

Reviewer #3: No
