# Supplementary material for: Treatment of adult neglected clubfoot with Ponseti method: Consecutive case series of thirty-three feet
Source: PLoS One. 2026 Sep 15;21(9):e0358249. doi: 10.1371/journal.pone.0358249 (PMC13577333; doi:10.1371/journal.pone.0358249)
Supplement: Supplementary file 4 [file pone.0358249.s018.docx]

Dear Editor-in-Chief,

## Thank you for considering the paper "**Treatment of adult neglected clubfoot with Ponseti Method: consecutive case series of thirty-three feet**", for publication in the *PLos One* and for allowing us to re-submit this revised manuscript.

The authors are grateful to the reviewers for the careful appraisal, positive comments, and helpful criticisms. Suggested changes have been addressed. We believe that the quality of the manuscript has been improved and hope that it now meets the quality required for publication in the *PLos One*.

A detailed point-by-point response is given in the response letter.

In addition, the Abstract has been revised (line 15) to explicitly state that this is the first reported case series of *previously untreated* adults with neglected clubfoot treated using the Ponseti method.

We are looking forward to your decision regarding the suitability of the revised version of this paper for the Journal.

Thank you very much in advance,

Reviewers' comments:

Reviewer's Responses to Questions

**Comments to the Author**

1. If the authors have adequately addressed your comments raised in a previous round of review and you feel that this manuscript is now acceptable for publication, you may indicate that here to bypass the “Comments to the Author” section, enter your conflict of interest statement in the “Confidential to Editor” section, and submit your "Accept" recommendation.

Reviewer #4: (No Response)

Reviewer #5: All comments have been addressed

Reviewer #6: (No Response)

Reviewer #7: (No Response)

2. Is the manuscript technically sound, and do the data support the conclusions?

The manuscript must describe a technically sound piece of scientific research with data that supports the conclusions. Experiments must have been conducted rigorously, with appropriate controls, replication, and sample sizes. The conclusions must be drawn appropriately based on the data presented.

Reviewer #4: Yes

Reviewer #5: Yes

Reviewer #6: No

Reviewer #7: Yes

3. Has the statistical analysis been performed appropriately and rigorously?

Reviewer #4: Yes

Reviewer #5: Yes

Reviewer #6: No

Reviewer #7: Yes

4. Have the authors made all data underlying the findings in their manuscript fully available?

The [PLOS Data policy](http://www.plosone.org/static/policies.action#sharing) requires authors to make all data underlying the findings described in their manuscript fully available without restriction, with rare exception (please refer to the Data Availability Statement in the manuscript PDF file). The data should be provided as part of the manuscript or its supporting information, or deposited to a public repository. For example, in addition to summary statistics, the data points behind means, medians and variance measures should be available. If there are restrictions on publicly sharing data—e.g. participant privacy or use of data from a third party—those must be specified.

Reviewer #4: Yes

Reviewer #5: Yes

Reviewer #6: Yes

Reviewer #7: Yes

5. Is the manuscript presented in an intelligible fashion and written in standard English?

PLOS ONE does not copyedit accepted manuscripts, so the language in submitted articles must be clear, correct, and unambiguous. Any typographical or grammatical errors should be corrected at revision, so please note any specific errors here.

Reviewer #4: Yes

Reviewer #5: Yes

Reviewer #6: Yes

Reviewer #7: Yes

6. Review Comments to the Author

Please use the space provided to explain your answers to the questions above. You may also include additional comments for the author, including concerns about dual publication, research ethics, or publication ethics. (Please upload your review as an attachment if it exceeds 20,000 characters)

Reviewer #4: The manuscript addresses an important and understudied topic. The data generally support the conclusions, but I have a few concerns that should be addressed before publication.

Response: We thank the reviewer for the positive evaluation of our manuscript and for the constructive comments. We have carefully considered each concern and addressed them individually below.

1.The criteria for performing additional bone surgery need to be clarified. It is not entirely clear from the methods section what threshold of residual deformity prompted surgical intervention. Also, the patient who received twenty-five casts despite the stated maximum of fifteen requires a brief explanation.

Response: We thank the reviewer for this valuable comment. We believe that these points are already addressed in the Methods section. The criteria for performing additional bone surgery are described in lines 127-130. Likewise, the reason why one patient received 25 cast changes is already explained in lines 117-119.

2.Some of the figures still show partially identifiable patient faces. These should be fully anonymized in line with journal policy.The discussion would benefit from a more direct comparison of outcomes and complications with Ponseti use in younger age groups, rather than primarily restating the present findings.

Response: We thank the reviewer for this valuable comment. The patient faces have now been fully anonymized in all figures, in accordance with the journal's policy. Regarding the Discussion, we added a comparison with younger age groups in lines 287-290, emphasizing that, unlike in adults, Ponseti highlighted the importance of achieving 70° of forefoot abduction before Achilles tenotomy in newborns. We believe this comparison adequately places our findings in the context of the established pediatric literature while maintaining the Discussion focused on the objectives of the present study.

3.Overall, the work is valuable and the method appears feasible. With the revisions noted above, the manuscript would be suitable for publication.

Response: We sincerely thank the reviewer for the positive assessment of our manuscript and for the constructive suggestions. We are pleased that the reviewer considers the manuscript suitable for publication after revision.

Reviewer #5: Thank you for the opportunity to review this manuscript. This is a clinically relevant study on a genuinely under-reported problem: the Ponseti method in adults with neglected, previously untreated congenital clubfoot. The cohort is difficult to assemble, the photographic documentation is excellent, and the clinical rationale is sound. My comments are deliberately focused on internal consistency: a set of numbers and cross-references that currently do not match across the abstract, text, tables and figures. These are all correctable and, once reconciled, the manuscript should be suitable for publication on technical grounds.

Response: We sincerely thank the reviewer for the positive and encouraging evaluation of our manuscript. We greatly appreciate the recognition of the clinical relevance of this study, the uniqueness of this patient cohort, and the quality of the photographic documentation. Following the reviewer's recommendation, we carefully re-examined the entire manuscript and systematically corrected all inconsistencies in numbers, cross-references, tables, figures, and text to ensure complete internal consistency throughout the manuscript.

1. Figure 1 does not reconcile with the narrative. It states "36 started treatment" and "15 not included in analysis," whereas the Methods and Abstract state that 30 started (36 selected, 6 waiting) and that 9 were excluded (5 short follow-up + 4 ongoing). Figure 1 also lists only 9 of the stated 15 and omits both the 6 patients "waiting to start" and the 2 who declined (lines 82-90). Please redraw so every node is labeled and the totals match the text.

Response: We thank the reviewer for identifying this inconsistency. We agree that the original flowchart did not fully reflect the patient selection process described in the manuscript. Figure 1 has been completely redesigned to include every step of patient selection and follow-up. The revised figure now explicitly shows: (1) the five patients excluded because of vascular risk factors; (2) the two patients who declined treatment; (3) the 36 patients selected for treatment; (4) the six patients who were still waiting to start treatment; (5) the 30 patients who actually started the Ponseti protocol; (6) the five patients with follow-up shorter than 12 months; (7) the four patients still undergoing treatment; and (8) the final cohort of 21 patients (33 feet) included in the analysis. Figure 1 has been revised to ensure complete numerical consistency.

2. The surgical-subgroup outcomes do not match Table 3. The text states "45.4% very good and 9.1% as regular, with no excellent results" (lines 227-230), which sums to only 54.5%. By Table 3, of the 11 feet that had additional bone surgery, 10 are graded very good and 1 regular (about 90.9% vs 9.1%). Please recompute and correct.

Response: We thank the reviewer for carefully identifying this numerical inconsistency. The reviewer is correct. The percentage of feet classified as very good among those requiring additional bone surgery was inadvertently reported as 45.4%, whereas the correct value is 90.9% (10 of 11 feet). The manuscript has been corrected accordingly in line 237. No other analyses or conclusions were affected by this correction.

3. Residual deformity figures conflict: Table 3 reports 58% of patients without residual deformity (i.e., 42% with), whereas the Discussion states deformities were present "in 50% of cases" (line 270). Please reconcile and state the denominator (per patient vs per foot).
Response: We thank the reviewer for identifying this ambiguity. The percentages reported in Table 3 were calculated using the foot, rather than the patient, as the denominator. At the last follow-up, 19 of 33 feet (57.6%) had no residual deformity, while 14 of 33 feet (42.4%) had residual deformity; 12 feet (36.4%) had mild deformity and two feet (6.1%) had moderate deformity. At the patient level, 10 of 21 patients (47.6%) had residual deformity in at least one foot. We have revised the Results, Discussion, and Table 3 to state the denominators explicitly and ensure consistency throughout the manuscript. In the Results section, it is better explained in lines 203-205 and in the Discussion section in lines 284-286.

4. The pre-treatment AOFAS value is 32 in the Abstract, Results (line 219) and Discussion, but 31 in the Table 3 summary row. Please make these consistent.
Response: We thank the reviewer for identifying this inconsistency. The reviewer is correct. The pre-treatment AOFAS value reported in the summary row of Table 3 was a typographical error. The correct mean pre-treatment AOFAS score is 32 (range, 8–50), consistent with the Abstract, Results, and Table 4. Table 3 has been corrected accordingly.

5. Cross-references are mismatched: "the degree of foot rotation (Fig 2)" (line 257) should cite Figure 3 (Figure 2 is flexibility), and "the patient (number 3 in Table 1)" (line 259) should cite Table 3 (Table 1 is the AOFAS rubric).

Response: We thank the reviewer for identifying these cross-reference errors. Both references have been corrected. The sentence referring to the degree of foot rotation now cites Figure 3, which presents the foot-rotation classification, rather than Figure 2, which presents the flexibility assessment. In addition, the reference to patient 3 has been corrected from Table 1 to Table 3, where the individual patient data are presented.

6. Flexibility terminology is not harmonized: it appears as "rigid/mild/moderate" (Table 3), "no/little/moderate" (Figure 2 legend) and "little or no" (Abstract). The "little to mild" change accepted in revision was not propagated to the Abstract or Figure 2. Please standardize the wording throughout.

Response: We thank the reviewer for identifying this inconsistency in terminology. We agree that flexibility classification should be uniform throughout the manuscript. The terminology has now been standardized across the Abstract, Figure 2, Table 3, Results, and Discussion. The flexibility categories are consistently reported as rigid, mild, and moderate, and all remaining references to "no" or "little" flexibility have been revised accordingly to ensure consistency throughout the manuscript.

7. The forefoot-abduction target is stated as 40 degrees (line 113) but appears as a "30-50 degrees" decision node in Figure 5. Please align the target between text and figure.
Response: We thank the reviewer for identifying this inconsistency. We agree that the text and Figure 5 should use the same definition. In our protocol, approximately 40° of forefoot abduction was the intended target, although an acceptable clinical range of 30° to 50° was used according to the correction achieved in each foot. The Methods section has been revised to clarify this range, and Figure 5 has been retained with the 30°–50° decision node to ensure consistency throughout the manuscript.

8. The pre-treatment tibiocalcaneal angle is reported with a standard deviation larger than its mean (46.75 +/- 52.95 degrees, line 196), which is implausible for an angular measurement and suggests a transcription error to be checked.

Response: We thank the reviewer for identifying this inconsistency. We carefully rechecked the original dataset and confirmed that the reported standard deviation resulted from a transcription error. The tibiocalcaneal angle values have been recalculated from the original data, and the correct standard deviation has been updated in the revised manuscript in line 207. This correction does not affect the statistical analyses or the conclusions of the study.

9. The pilon impaction fracture is attributed to two feet in the text (patient 3 right, patient 20 left, line 185) but appears documented for patient 3 only in Figure 6. Please confirm and align.

Response: We thank the reviewer for this important observation. We confirmed that pilon impaction fractures occurred in two feet: the right foot of patient 3 and the left foot of patient 20. Figure 6 was intended to illustrate only the representative case of patient 3. To avoid any misunderstanding, the text in lines 193-194 has been revised to explicitly state that Figure 6 depicts only the right foot of patient 3, while a similar pilon impaction fracture also occurred in the left foot of patient 20.

10. Two ethics identifiers appear: the IRB approval number 1.668.854 in the submission metadata, and CAAE 53452216.9.3001.5463 in the manuscript (line 330). If these refer to the same approval, please state both consistently in the Methods.
Response: We thank the reviewer for this observation. The IRB approval number (1.668.854) and the CAAE registration number (53452216.9.3001.5463) refer to the same ethics approval. To avoid any ambiguity, the manuscript has been revised to match the submission metadata.

11. The in-article Data Availability statement (lines 313-316) reads "available from the corresponding author upon reasonable request," which is not consistent with the submission-form statement and the attached S1 Dataset. Please harmonize the wording to reflect that the data are deposited in the Supporting Information.
Response: We thank the reviewer for this observation. The Data Availability Statement has been revised to indicate that the datasets are available in the Supporting Information (S1 Dataset), ensuring consistency with the submission.

12. The odds ratio for residual deformity by sex (line 212) is printed as "OR = 20.0; IC 95%, 1;39-301.00": "IC" should read "CI" and "1;39" appears to be a typo for 1.39. A few residual typographical errors remain (e.g., "notfollow" line 192; "s ex" line 23; "similars work" line 309).

Response: We thank the reviewer for the careful proofreading of the manuscript. The typographical errors identified by the reviewer have been corrected, including changing "IC" to "CI", correcting the confidence interval from "1;39" to "1.39", and revising the remaining typographical errors (e.g., "notfollow", "s ex", and "similars work"). The manuscript has been carefully proofread to correct these and other minor typographical inconsistencies.

13. The 11 feet / 7 patients relationship is correct but reads as contradictory on first pass; a short bridging clause when the surgical subgroup is introduced would help the reader.

Response: We thank the reviewer for this helpful suggestion. To improve clarity, the text has been revised in lines 176-177 to specify that the 11 feet requiring additional bone surgery belonged to seven patients, including four with bilateral involvement, thereby avoiding any apparent discrepancy between the number of feet and patients.

14. References: reference 13 (Pirani et al., MRI study) is dated 2021, but the cited volume corresponds to 2001; and reference 17 is attributed in the text to the "American Pediatric Society" (line 85), whereas the source is the American Academy of Pediatrics. Please verify both.

Response: We thank the reviewer for this careful observation. We verified both references and corrected the identified errors. Specifically, the publication year of Reference 13 has been corrected, and the in-text citation (line 86) has been revised to correctly refer to the American Academy of Pediatrics. The reference list has also been carefully reviewed for consistency.

Overall this is a worthwhile study with clear clinical merit. The points above are corrections to existing content rather than requests for new data or analyses, so my recommendation is Minor Revision. I would be glad to look at the revised version.

Response: We sincerely thank the reviewer for the thorough and constructive evaluation of our manuscript. We greatly appreciate the positive assessment of the clinical relevance of our study and the recommendation for minor revision. We have carefully addressed all comments and revised the manuscript accordingly. We are grateful for the reviewer's valuable suggestions, which have substantially improved the clarity and consistency of the manuscript.

Reviewer #6: This manuscript reports the treatment outcomes of neglected congenital talipes equinovarus (CTEV) in adults—a topic with limited representation in the literature. While the study addresses an important clinical gap, the presentation is disorganized and lacks the logical flow expected of a scientific paper. My detailed comments are as follows:

Response: We sincerely thank the reviewer for the thoughtful evaluation of our manuscript and for recognizing the clinical relevance of this study and the limited evidence available on the treatment of neglected congenital talipes equinovarus in adults. We appreciate the reviewer's comments regarding the organization and logical flow of the manuscript. Accordingly, we have carefully revised the manuscript to improve its structure, clarity, and overall readability, while addressing each of the specific comments provided below.

Title
• The title suggests that the Ponseti method was the sole treatment modality. However, additional surgical procedures (beyond tendoachilles [TA] tenotomy) were performed. The title should be revised to reflect the full scope of interventions.

Response: We thank the reviewer for this thoughtful comment. We acknowledge that additional bone procedures were required in a subset of feet with residual deformity following the Ponseti protocol. However, we chose to retain the current title because all patients were initially treated using the Ponseti method, which constituted the primary treatment strategy in this series. Additional bone procedures were performed only in selected feet when residual deformities persisted after completion of the Ponseti protocol. Therefore, we believe the current title accurately reflects the principal focus of the study.

Abstract
• As with the title, the abstract states Ponseti as the only treatment objective, yet other procedures were included when correction was incomplete.

Response: We thank the reviewer for this valuable comment. To avoid any ambiguity, the Abstract has been revised to explicitly state that all patients were initially treated using the Ponseti method, whereas additional bone surgery was performed only in selected feet with residual deformity after serial cast changes and Achilles tenotomy.

• The number of patients (30) does not match the figure reported in the Introduction (43). This inconsistency must be corrected.

Response: We thank the reviewer for this comment. The numbers refer to different stages of patient selection: 43 patients were initially evaluated, whereas 30 patients started treatment with the Ponseti method. To avoid any misunderstanding, Figure 1 has been completely revised to clearly illustrate the patient-selection process, including all exclusions, patients who declined treatment, those awaiting treatment initiation, and the final study cohort.

• Statistical analysis is inadequately described. The specific correlation test used should be identified, as well as the test applied for pre and post AOFAS score comparisons.

Response: We thank the reviewer for this important comment. The Statistical Analysis section has been revised to specify that Spearman’s rank correlation coefficient was used for correlation analyses and that the Wilcoxon signed-rank test was used to compare pre- and post-treatment AOFAS scores.

• The conclusion is misleading, as it implies Ponseti alone is sufficient for neglected adult CTEV. It should be clarified to acknowledge the role of adjunctive procedures.
Response: We thank the reviewer for this valuable comment. The Conclusion has been revised to clarify that the Ponseti method was used as the initial treatment in all patients, whereas additional bone surgery was performed only in selected feet with residual deformities after completion of the Ponseti protocol. The revised wording more accurately reflects the treatment strategy used in this study.

Introduction
• The description of the Ponseti method is overly brief and insufficient, given its central role in the study.

Response: We thank the reviewer for this comment. The Introduction was intended to provide only a brief overview of the Ponseti method. The complete treatment protocol used in this study is described in detail in the Methods section, including the casting technique, Achilles tenotomy, and the indications for additional bone surgery.

• Key terminology such as “foot flexibility” and “foot rotation” should be introduced here, supported by relevant literature, to justify their use as outcome measures.
Methods
Response: We thank the reviewer for this valuable comment. The definitions of foot flexibility and foot rotation, as well as their assessment, are described in the Methods section. These variables were included because, during the treatment of this series of adult patients, we observed that they were clinically relevant indicators of deformity severity, correctability, and treatment outcome. To the best of our knowledge, these parameters have not been previously and systematically evaluated as outcome-related variables in studies of the Ponseti method, particularly in adults.

• Patient numbers are inconsistent between the Abstract, Introduction, and flowchart.
Response: We thank the reviewer for this valuable comment. The apparent discrepancy resulted from the description of different stages of patient selection. To eliminate any ambiguity, Figure 1 has been completely revised to clearly illustrate the entire recruitment and inclusion process. In addition, the Abstract, Introduction, and Methods have been revised to ensure that the patient numbers are fully consistent throughout the manuscript.

• The assessment of foot flexibility appears subjective, with limited validity beyond stratifying subjects into groups. The rationale and limitations should be explained.
Response: We thank the reviewer for this valuable comment. We agree that the assessment of foot flexibility is based on clinical examination and therefore has an inherent degree of subjectivity. However, this parameter was included because, during the treatment of this series, we observed that it was clinically useful for estimating deformity correctability and stratifying patients according to treatment difficulty. To address the reviewer's concern, we have added this point as a limitation in the Discussion. See lines 264-267.

• Foot rotation assessment lacks standardization. The methodology for frontal photographs (stance, device used, distance from subject) must be described in detail.
Response: We thank the reviewer for this valuable comment. The description of the foot rotation assessment has been expanded to provide a more standardized and detailed methodology. Specifically, the revised Figure 3 legend now describes the patient positioning during image acquisition, including weight-bearing, static stance, and the patellae facing directly forward without lower-limb rotation, as well as the anatomical landmarks used to measure the angle. These modifications have been incorporated in lines 428-432 of the revised manuscript.

• The TA tenotomy procedure requires clarification: was it open or percutaneous? In adults, Z lengthening is often necessary. Performing the procedure under local anesthesia/sedation alone is unusual and should be justified.

Response: We thank the reviewer for this valuable comment. The Methods section has been revised to clarify that the Achilles tenotomy was performed percutaneously. We also clarified the anesthetic technique, specifying that only the first five patients underwent the procedure under sedation associated with local anesthesia, whereas all subsequent patients underwent spinal anesthesia. The only exception was the patient with myelomeningocele, who underwent percutaneous Achilles tenotomy under local anesthesia without sedation in the outpatient clinic. See lines 120-124.

• For all assessments and interventions, the number of surgeons/assessors must be stated, and inter observer reliability should be addressed where appropriate.
Response: We thank the reviewer for this valuable comment. The Methods section has been revised to clarify that all clinical assessments, treatment decisions, and surgical procedures were performed by the senior author, thereby ensuring consistency in patient evaluation and treatment throughout the study. As all assessments were performed by a single observer, interobserver reliability was not applicable and therefore was not evaluated. This information has been added to the Methods section (lines 145-146 of the revised manuscript).

• In bilateral cases, the method of data analysis should be specified (e.g., worse foot only, mean/median of both).

Response: We thank the reviewer for this valuable comment. As shown in Table 3, the outcome analyses were performed per foot (33 feet), rather than per patient. To make this clearer, this has been specified in the revised Methods section. This information has been added to the Methods section (lines 142-143 of the revised manuscript).

• Statistical methods should begin with a normality test, especially given the small sample size. This dictates whether mean or median values are appropriate, and whether parametric or non-parametric tests should be applied.

Response: We thank the reviewer for this important comment. The Statistical Analysis section has been revised to explicitly identify all statistical tests used in the study. Specifically, Spearman's rank correlation coefficient was used for correlation analyses, the Wilcoxon signed-rank test for comparison of pre- and post-treatment AOFAS scores, the Mann-Whitney U and Kruskal-Wallis nonparametric tests for group comparisons, and the chi-square or Fisher's exact test, as appropriate, for comparison in categorical variables.

Results
• Results should begin with a clear description of the study population. Table 3 can summarize this; Table 2 is not useful and may be removed.

Response: We thank the reviewer for this valuable comment. We respectfully believe that the current organization of the Results section provides a logical presentation of the study population and subsequent findings. Therefore, we have chosen to maintain the current structure. Likewise, Table 2 was retained because it provides relevant individual patient information that complements the summarized data in Table 3.

• Table 4 does not conform to scientific reporting standards. Analyses should specify the number of subjects (N), the statistical test used (e.g., t test, ANOVA), and p values. For correlation analyses, graphical representation (scatter plots) is more appropriate.
Response: We thank the reviewer for this valuable comment. We agree that the statistical methods should be clearly identified. Accordingly, the statistical tests used have been specified in the Methods section. We respectfully chose to retain Table 4 in its current format because it provides a concise summary of all statistical analyses performed. We believe that replacing the table with multiple scatter plots would substantially increase the length of the manuscript without improving the overall presentation of the results.

Discussion
• The discussion is superficial and lacks organization.

Response: We thank the reviewer for this valuable comment. We respectfully believe that the Discussion adequately addresses the principal findings of the study, compares them with the available literature, discusses their clinical implications, and acknowledges the study limitations. Therefore, we have chosen to maintain its overall organization.

• Study limitations—many of which are methodological—should be explicitly acknowledged and discussed.

Response: We thank the reviewer for this valuable comment. The Discussion has been revised to more explicitly acknowledge the methodological limitations of the study. In addition to the previously described limitations (lines 312-313), we have also included the subjective assessment of foot flexibility as a limitation of the study.

• Greater depth is needed to contextualize findings within existing literature (comparison with neglected cases in children can be used), highlight the challenges of treating neglected adult CTEV, and clarify the role of Ponseti relative to adjunctive procedures.

Response: We thank the reviewer for this valuable comment. We agree that comparisons with the pediatric literature are useful when discussing the principles of the Ponseti method. Accordingly, we expanded the Discussion by adding a comparison with younger age groups (lines 287-290), emphasizing that, unlike in adults, Ponseti highlighted the importance of achieving 70° of forefoot abduction before Achilles tenotomy in newborns. We also added the statement that some degree of bone remodeling may occur in adult patients, although to a lesser extent than in children (lines 308-309). However, we believe that direct comparison of treatment outcomes between children and adults with neglected clubfoot is of limited value because of the substantial differences in skeletal maturity, soft-tissue stiffness, and remodeling potential. Therefore, we focused our discussion primarily on the available literature concerning neglected clubfoot in adults and on the specific challenges of treating this population. In addition, we clarified throughout the revised manuscript that all patients were initially treated using the Ponseti method, with additional bone surgery reserved only for feet that were not fully corrected after serial cast changes and Achilles tenotomy.

Reviewer #7: Review of PONE

PONE-D-25-14281
"Treatment of adult neglected clubfoot with Ponseti Method: consecutive case series of thirty-three feet"

Congratulations, it is a very important paper and deserves to be published.
Below are just a few points that can be improved.

Response: We sincerely thank reviewer for the positive assessment of our manuscript and for the constructive suggestions. We greatly appreciate the recognition of the importance of this study and have carefully addressed each comment below.

Discussion
239 - and the “shortage of qualified surgeons” – this is a dangerous sentence. Lots of surgeons do think that patient could loose walking habilities being so much time without walking, and are afraid to do so, but not because of expertise…

Response: We thank the reviewer for this valuable comment. To avoid this misunderstanding, we replaced the word “qualified” with “specialized” .

251 – “In our study, for the first time,” – this repeating for the first time is not academically appropriated. This is to be judged by the readers. Please take out these so repeated and inadequate terms.

Response: We thank the reviewer for this valuable comment. We agree that such statements are better left to the readers' interpretation. Therefore, we removed the repeated expressions referring to "for the first time" throughout the manuscript.

289 – “stability in neglected clubfoot patients” – It would be nice to take out the neglected term, considered pejorative and negative – better to substitute for non treated adults

Response: We thank the reviewer for this valuable comment. We agree that the term "neglected" may carry an unintended negative connotation in this context. Therefore, we replaced it with "non-treated adults" in the revised manuscript.

It would be nice to have a point in the discussion about bony remodeling, based in what you have seen in the clinical and radiographic aspects of your patients. This is to make a correspondence between Pirani´s 2001 MRI article, this
Nogueira MP, Amaral DT. How much remodelling is possible in a clubfoot treatment? Magnetic resonance imaging study in a 7-year-old child. J Limb Lengthen Reconstr. 2018;4(1):49-54.
And yours.

Response: We thank the reviewer for this valuable suggestion. We have addressed this point by adding a statement to the Discussion acknowledging that some degree of bone remodeling may occur in adult patients, although to a lesser extent than that reported in children. This addition has been incorporated in lines 308-309.
