# Supplementary material for: Treatment of adult neglected clubfoot with Ponseti method: Consecutive case series of thirty-three feet
Source: PLoS One. 2026 Sep 15;21(9):e0358249. doi: 10.1371/journal.pone.0358249 (PMC13577333; doi:10.1371/journal.pone.0358249)
Supplement: Peer review history

# Peer review: Decision Letter 0

**DOI:** 10.1371/journal.pone.0358249.r001

**Authors:** Seyed Arman Moein

2 Nov 2025

Dear Dr. Haje,

Please submit your revised manuscript by Dec 17 2025 11:59PM. If you will need more time than this to complete your revisions, please reply to this message or contact the journal office at plosone@plos.org. When you're ready to submit your revision, log on to https://www.editorialmanager.com/pone/ and select the 'Submissions Needing Revision' folder to locate your manuscript file.

- A rebuttal letter that responds to each point raised by the academic editor and reviewer(s). You should upload this letter as a separate file labeled 'Response to Reviewers'.
- A marked-up copy of your manuscript that highlights changes made to the original version. You should upload this as a separate file labeled 'Revised Manuscript with Track Changes'.
- An unmarked version of your revised paper without tracked changes. You should upload this as a separate file labeled 'Manuscript'.

If you would like to make changes to your financial disclosure, please include your updated statement in your cover letter. Guidelines for resubmitting your figure files are available below the reviewer comments at the end of this letter.

If applicable, we recommend that you deposit your laboratory protocols in protocols.io to enhance the reproducibility of your results. Protocols.io assigns your protocol its own identifier (DOI) so that it can be cited independently in the future. For instructions see: https://journals.plos.org/plosone/s/submission-guidelines#loc-laboratory-protocols. Additionally, PLOS ONE offers an option for publishing peer-reviewed Lab Protocol articles, which describe protocols hosted on protocols.io. Read more information on sharing protocols at https://plos.org/protocols?utm_medium=editorial-email&utm_source=authorletters&utm_campaign=protocols.

We look forward to receiving your revised manuscript.

Kind regards,

Seyed Arman Moein, M.D.

Guest Editor

PLOS ONE

Journal Requirements:

When submitting your revision, we need you to address these additional requirements.

1. Please ensure that your manuscript meets PLOS ONE's style requirements, including those for file naming. The PLOS ONE style templates can be found at

https://journals.plos.org/plosone/s/file?id=wjVg/PLOSOne_formatting_sample_main_body.pdf and

https://journals.plos.org/plosone/s/file?id=ba62/PLOSOne_formatting_sample_title_authors_affiliations.pdf

2. We noted in your submission details that a portion of your manuscript may have been presented or published elsewhere. “https://ssrn.com/abstract=4986324 or http://dx.doi.org/10.2139/ssrn.4986324” Please clarify whether this publication was peer-reviewed and formally published. If this work was previously peer-reviewed and published, in the cover letter please provide the reason that this work does not constitute dual publication and should be included in the current manuscript.

3. Your ethics statement should only appear in the Methods section of your manuscript. If your ethics statement is written in any section besides the Methods, please move it to the Methods section and delete it from any other section. Please ensure that your ethics statement is included in your manuscript, as the ethics statement entered into the online submission form will not be published alongside your manuscript.

4. We note that Figure 1 - 8 includes an image of a patient in the study.

As per the PLOS ONE policy (http://journals.plos.org/plosone/s/submission-guidelines#loc-human-subjects-research) on papers that include identifying, or potentially identifying, information, the individual(s) or parent(s)/guardian(s) must be informed of the terms of the PLOS open-access (CC-BY) license and provide specific permission for publication of these details under the terms of this license. Please download the Consent Form for Publication in a PLOS Journal (http://journals.plos.org/plosone/s/file?id=8ce6/plos-consent-form-english.pdf). The signed consent form should not be submitted with the manuscript, but should be securely filed in the individual's case notes. Please amend the methods section and ethics statement of the manuscript to explicitly state that the patient/participant has provided consent for publication: “The individual in this manuscript has given written informed consent (as outlined in PLOS consent form) to publish these case details”.

If you are unable to obtain consent from the subject of the photograph, you will need to remove the figure and any other textual identifying information or case descriptions for this individual.

5. If the reviewer comments include a recommendation to cite specific previously published works, please review and evaluate these publications to determine whether they are relevant and should be cited. There is no requirement to cite these works unless the editor has indicated otherwise.

Additional Editor Comments:

Experts in this field have reviewed your article. There have been some concerns regarding the method and results of your study outlined by the reviewers. Please answer them carefully, in a point-by-point manner. Moreover, your article seems to require English editing services.

[Note: HTML markup is below. Please do not edit.]

Reviewers' comments:

Reviewer's Responses to Questions

**Comments to the Author**

1. Is the manuscript technically sound, and do the data support the conclusions?

Reviewer #1: Yes

Reviewer #2: Partly

Reviewer #3: Yes

**********

2. Has the statistical analysis been performed appropriately and rigorously?

Reviewer #1: I Don't Know

Reviewer #2: I Don't Know

Reviewer #3: Yes

**********

3. Have the authors made all data underlying the findings in their manuscript fully available?

The PLOS Data policy

Reviewer #1: Yes

Reviewer #2: Yes

Reviewer #3: Yes

**********

4. Is the manuscript presented in an intelligible fashion and written in standard English?

Reviewer #1: Yes

Reviewer #2: No

Reviewer #3: Yes

**********

Reviewer #1: Dear Editor,

Thank you for the opportunity to review this manuscript. The authors present an important and timely study on a challenging clinical problem. The use of the Ponseti method for neglected clubfoot in an adult population is a valuable area of research, and this case series is a significant contribution. The manuscript is well-written, but I have several comments and questions that the authors should address to strengthen their work.

My review is organized by section below.

Introduction

The introduction effectively establishes the clinical problem. The literature review is logical and concise, providing a strong justification for the study, and the objectives are clearly stated.

• Minor Suggestion: The authors use the term "medium number of casts." I would recommend replacing this with the more standard scientific terminology of "mean" or "average number of casts."

________________________________________

Methods

This section raises several important questions regarding patient selection and methodological clarity.

• Exclusion Criteria: The rationale for excluding patients with a history of thrombosis requires clarification. Could these patients not be managed with prophylactic anticoagulation, similar to the protocol used for other high-risk patients in the study?

• Anticoagulation Guidelines: The manuscript should cite the specific guidelines or criteria used for prescribing anticoagulants to patients who were overweight or over 50 years old.

• Flexibility Assessment: The assessment of pre-treatment flexibility is described qualitatively ('little', 'moderate', 'rigid'). The authors should discuss why a quantitative classification system, such as the DeMilio classification for clubfoot, was not used to provide a more objective and reproducible measure.

• Neuromuscular Conditions: The manuscript needs to be more explicit about the presence of associated neuromuscular conditions. While one case of myelomeningocele is mentioned, it is unclear if other conditions like arthrogryposis were present in the cohort or were part of the exclusion criteria.

• Radiographic Analysis: The pre-operative radiographic analysis did not include an assessment for flat-top talus. Given that this finding can probably predict the need for more extensive surgery beyond an Achilles tenotomy, its presence or absence is valuable information that should be reported and discussed.

________________________________________

Results

The presentation of the results could be enhanced with more quantitative detail.

• Defining Outcomes: The terms "relapse" and "residual deformity" lack quantitative definition. The authors should specify how these were measured or graded. Was there a standardized method to quantify the degree of remaining or recurrent deformity?

• Surgical Decision-Making: The decision-making process for performing additional surgery after casting and tenotomy is unclear. The results table presents an apparent contradiction where some patients with 'mild' residual deformity underwent surgery, while another with a 'moderate' deformity (Patient 11) did not. The criteria for this crucial clinical decision must be explicitly detailed.

________________________________________

Discussion

The discussion is well-grounded but would benefit from addressing the study's limitations more directly.

• Impact of Sample Size: The discussion would be strengthened by a more thorough examination of the study's limitations, particularly the small sample size and its potential impact on the statistical power and generalizability of the findings. While acknowledging the difficulty of recruiting such a cohort is fair, the implications of this limitation must be addressed.

• Lack of a Comparative Group: The authors should also discuss the lack of a comparative group treated with standard surgical methods, which is a key limitation when evaluating the efficacy of the proposed treatment.

________________________________________

Suggestion for Future Research

The authors have laid the groundwork for a valuable future study. Their current cohort consists of previously untreated patients. However, many patients in this age group may have undergone procedures like posteromedial release in the past and now present with residual or recurrent deformities. Since this study demonstrates the feasibility of the Ponseti method even in rigid feet, applying a similar protocol to this post-surgical population would be a logical and impactful next step.

In summary, this is a noteworthy paper that, with revision, will be a strong addition to the literature. I look forward to seeing a revised version that addresses these points.

Reviewer #2: Dear Authors,

Your manuscript provides valuable insights into the treatment of neglected clubfoot in adults. However, I believe the paper requires major revisions as outlined below:

The manuscript contains several instances of language use that are not suitable for a scientific paper. I recommend seeking assistance from a native English speaker or using AI tools only for grammar checking and paraphrasing.

The introduction does not follow a standard academic structure and lacks logical flow. For instance, the content in lines 64–65 would fit better in the first paragraph, while lines 57–63 could be used to describe current treatment methods for neglected clubfoot in adults. I strongly suggest reorganizing the introduction into 3–4 coherent paragraphs following the conventional structure (background, problem statement, existing knowledge, and study aim).

Lines 141–142: The sentence “All patients received a pair of sneakers donated by the main author, as the first sneaker in life after the removal of the last cast” is not appropriate and ethical for inclusion in a scientific paper and should be removed.

The results section reports numerous p-values but lacks corresponding means and standard deviations. Please include these descriptive statistics where applicable.

Line 175: The term “Medium” is not standard in statistical reporting and should be replaced with the correct term (e.g., “median” or “mean,” depending on the intended meaning).

The discussion section primarily reiterates the authors’ findings without adequately comparing them to existing literature or alternative treatment methods. Please revise this section to include a critical comparison with relevant studies.

Some of the included patient images show faces that are partially identifiable. This is unprofessional and raises ethical concerns. All identifiable features must be removed or blurred in accordance with ethical publishing standards.

Reviewer #3: Dear authors, thank you for your manuscript. Some concerns and suggestions for improvement are in order, and I have laid them out line by line:

Introduction

Line 58: Please include a few sentences explaining the Ponseti technique, particularly emphasizing its principles and rationale.

Method

1. Please specify the study design. Was it a prospective cohort study or another type?

2. Including a flowchart illustrating the patient selection and inclusion process (from 43 initial patients to the final 21) would enhance clarity. If this is not feasible, please elaborate more. Take Line 100 for instance, six patients were “waiting for treatment”, it is unclear what they were waiting for and why they were excluded.

3. Some grammatical corrections are needed. Specifically lines 121, 183, and 249-251.

4. Line 125: The manuscript states that up to 15 cast changes were performed. Considering that each cast was applied for seven days, this equates to approximately 3.5 months of serial casting which raises concerns of knee stiffness due to prolonged immobilization. Please clarify.

5. Line 130: The inclusion of a patient with myelomeningocele is questionable. Managing clubfoot in such patients is challenging, often requiring multiple tendon transfers or osteotomies to achieve a plantigrade foot. Serial casting in an adult with myelomeningocele is unlikely to be effective.

6. Line 135: Please clarify the criteria used to determine when patients required surgical intervention. How many cast changes were performed before surgery was indicated?

7. Several patients reportedly had bilateral deformities. What was your protocol for them? Were casts applied simultaneously to both feet, or sequentially?

8. While the postoperative protocol is described, there is no mention of the post-casting protocol. Please include details such as the need for bracing, type of brace, and duration or schedule of use.

Results

1. Line 175: You mention a median of 11.6 cast changes (range 3–25) before Achilles tenotomy, yet earlier you stated a maximum of 15 cast changes. Please clarify this discrepancy.

2. Lines 175, 185: Replace the term “grade” with “level” throughout for consistency.

3. Line 174, Table 3: Replace the term “little” with “mild” for better consistency.

4. Line 174, Table 3: Define the categories of mild, moderate, and rigid flexibility in the Methods section.

5. Line 200, Table 3: Clarify how you defined mild, moderate, and severe deformities (equinus, cavus, adduction). This should also be described in the Methods section.

6. Lines 210–234: For clarity and better visualization, present the correlation findings in a table format.

7. The Results mention complications, but there is no information on possible knee joint issues after long-term casting. Please clarify whether any stiffness, pain, or limitation occurred in the knees after cast removal, given the prolonged semi-flexed position during treatment. If no data available, please mention this in the limitations parts.

Discussion

1. Line 239: Begin the discussion by clearly emphasizing the key findings of your study.

2. Lines 247–251: The Ponseti method can be technically challenging even in neonates due to the risk of circulatory or skin complications. Numerous reports highlight these risks. Therefore, the claim that “the Ponseti method probably has fewer circulatory complications than talectomy or external fixator devices and can be used in older populations” is not sufficiently supported given the limited number of cases and potential selection bias.

3. Lines 263–269: Casting may not replace surgery for neglected clubfoot; however, it may reduce the magnitude of surgery required. Discuss this potential benefit in your Discussion. You might also compare complication rates and technical demands of complex surgical procedures versus your approach.

4. Lines 275–276: The statement implying that casting yields better outcomes than surgery could be misleading due to selection bias. Such a claim would require further studies, ideally randomized controlled trials (RCTs).

5. Line 281: Details regarding the brace type, duration, and use should be moved to the Methods section.

6. Line 294: Include a dedicated section outlining the limitations of your study. The bare minimum is to mention the small sample size, selection bias, and lack of long-term follow-up. Add suggestions for future research.

References

Many of the cited studies are outdated. Please update the reference list with more recent and relevant publications.

Overall, this study presents an interesting and innovative adaptation of the Ponseti method. With comprehensive revisions to methodology, data presentation, and language, it could make a valuable contribution to the literature.

**********

what does this mean?). If published, this will include your full peer review and any attached files.

If you choose “no”, your identity will remain anonymous but your review may still be made public.

**Do you want your identity to be public for this peer review?**  For information about this choice, including consent withdrawal, please see our Privacy Policy

Reviewer #1: No

Reviewer #2: **Yes:** Reza Niakan

Reviewer #3: No

**********

[NOTE: If reviewer comments were submitted as an attachment file, they will be attached to this email and accessible via the submission site. Please log into your account, locate the manuscript record, and check for the action link "View Attachments". If this link does not appear, there are no attachment files.]

To ensure your figures meet our technical requirements, please review our figure guidelines: https://journals.plos.org/plosone/s/figures

You may also use PLOS’s free figure tool, NAAS, to help you prepare publication quality figures: https://journals.plos.org/plosone/s/figures#loc-tools-for-figure-preparation.

NAAS will assess whether your figures meet our technical requirements by comparing each figure against our figure specifications.

# Author comment: Author response to Decision Letter 1

**DOI:** 10.1371/journal.pone.0358249.r002

22 Dec 2025

Dear Editor-in-Chief,

Thank you for considering the paper "Treatment of adult neglected clubfoot with Ponseti Method: consecutive case series of thirty-three feet", for publication in the PLos One and for allowing us to re-submit this revised manuscript.

The authors are grateful to the reviewers for the careful appraisal, positive comments, and helpful criticisms. Suggested changes have been addressed. We believe that the quality of the manuscript has been improved and hope that it now meets the quality required for publication in the PLos One.

A detailed point-by-point response is attached.

We are looking forward to your decision regarding the suitability of the revised version of this paper for the Journal.

Thank you very much in advance,

Attachment Submitted filename: Authors response_R1.docx

# Peer review: Decision Letter 1

**DOI:** 10.1371/journal.pone.0358249.r003

**Authors:** Raffaele Vitiello

29 Jun 2026

Dear Dr. Haje,

Thank you for submitting your manuscript to PLOS ONE. After careful consideration, we feel that it has merit but does not fully meet PLOS ONE’s publication criteria as it currently stands. Therefore, we invite you to submit a revised version of the manuscript that addresses the points raised during the review process.

Please submit your revised manuscript by Aug 13 2026 11:59PM. If you will need more time than this to complete your revisions, please reply to this message or contact the journal office at plosone@plos.org. When you're ready to submit your revision, log on to https://www.editorialmanager.com/pone/ and select the 'Submissions Needing Revision' folder to locate your manuscript file.

- A letter that responds to each point raised by the academic editor and reviewer(s). You should upload this letter as a separate file labeled 'Response to Reviewers'.
- A marked-up copy of your manuscript that highlights changes made to the original version. You should upload this as a separate file labeled 'Revised Manuscript with Track Changes'.
- An unmarked version of your revised paper without tracked changes. You should upload this as a separate file labeled 'Manuscript'.

If you would like to make changes to your financial disclosure, please include your updated statement in your cover letter. Guidelines for resubmitting your figure files are available below the reviewer comments at the end of this letter.

If applicable, we recommend that you deposit your laboratory protocols in protocols.io to enhance the reproducibility of your results. Protocols.io assigns your protocol its own identifier (DOI) so that it can be cited independently in the future. For instructions see: https://journals.plos.org/plosone/s/submission-guidelines#loc-laboratory-protocols. Additionally, PLOS ONE offers an option for publishing peer-reviewed Lab Protocol articles, which describe protocols hosted on protocols.io. Read more information on sharing protocols at https://plos.org/protocols?utm_medium=editorial-email&utm_source=authorletters&utm_campaign=protocols.

As the corresponding author, your ORCID iD is verified in the submission system and will appear in the published article. PLOS supports the use of ORCID, and we encourage all coauthors to register for an ORCID iD and use it as well. Please encourage your coauthors to verify their ORCID iD within the submission system before final acceptance, as unverified ORCID iDs will not appear in the published article. *Only*  the individual author can complete the verification step; PLOS staff *cannot*  verify ORCID iDs on behalf of authors.

We look forward to receiving your revised manuscript.

Kind regards,

Raffaele Vitiello

Academic Editor

PLOS One

Journal Requirements:

If the reviewer comments include a recommendation to cite specific previously published works, please review and evaluate these publications to determine whether they are relevant and should be cited. There is no requirement to cite these works unless the editor has indicated otherwise.

[Note: HTML markup is below. Please do not edit.]

Reviewers' comments:

Reviewer's Responses to Questions

**Comments to the Author**

Reviewer #4: (No Response)

Reviewer #5: All comments have been addressed

Reviewer #6: (No Response)

Reviewer #7: (No Response)

**********

2. Is the manuscript technically sound, and do the data support the conclusions?

Reviewer #4: Yes

Reviewer #5: Yes

Reviewer #6: No

Reviewer #7: Yes

**********

3. Has the statistical analysis been performed appropriately and rigorously?

Reviewer #4: Yes

Reviewer #5: Yes

Reviewer #6: No

Reviewer #7: Yes

**********

4. Have the authors made all data underlying the findings in their manuscript fully available?

The PLOS Data policy

Reviewer #4: Yes

Reviewer #5: Yes

Reviewer #6: Yes

Reviewer #7: Yes

**********

5. Is the manuscript presented in an intelligible fashion and written in standard English?

Reviewer #4: Yes

Reviewer #5: Yes

Reviewer #6: Yes

Reviewer #7: Yes

**********

Reviewer #4: The manuscript addresses an important and understudied topic. The data generally support the conclusions, but I have a few concerns that should be addressed before publication.

The criteria for performing additional bone surgery need to be clarified. It is not entirely clear from the methods section what threshold of residual deformity prompted surgical intervention. Also, the patient who received twenty-five casts despite the stated maximum of fifteen requires a brief explanation.

Some of the figures still show partially identifiable patient faces. These should be fully anonymized in line with journal policy.The discussion would benefit from a more direct comparison of outcomes and complications with Ponseti use in younger age groups, rather than primarily restating the present findings.

Overall, the work is valuable and the method appears feasible. With the revisions noted above, the manuscript would be suitable for publication.

Reviewer #5: Thank you for the opportunity to review this manuscript. This is a clinically relevant study on a genuinely under-reported problem: the Ponseti method in adults with neglected, previously untreated congenital clubfoot. The cohort is difficult to assemble, the photographic documentation is excellent, and the clinical rationale is sound. My comments are deliberately focused on internal consistency: a set of numbers and cross-references that currently do not match across the abstract, text, tables and figures. These are all correctable and, once reconciled, the manuscript should be suitable for publication on technical grounds.

1. Figure 1 does not reconcile with the narrative. It states "36 started treatment" and "15 not included in analysis," whereas the Methods and Abstract state that 30 started (36 selected, 6 waiting) and that 9 were excluded (5 short follow-up + 4 ongoing). Figure 1 also lists only 9 of the stated 15 and omits both the 6 patients "waiting to start" and the 2 who declined (lines 82-90). Please redraw so every node is labeled and the totals match the text.

2. The surgical-subgroup outcomes do not match Table 3. The text states "45.4% very good and 9.1% as regular, with no excellent results" (lines 227-230), which sums to only 54.5%. By Table 3, of the 11 feet that had additional bone surgery, 10 are graded very good and 1 regular (about 90.9% vs 9.1%). Please recompute and correct.

3. Residual deformity figures conflict: Table 3 reports 58% of patients without residual deformity (i.e., 42% with), whereas the Discussion states deformities were present "in 50% of cases" (line 270). Please reconcile and state the denominator (per patient vs per foot).

4. The pre-treatment AOFAS value is 32 in the Abstract, Results (line 219) and Discussion, but 31 in the Table 3 summary row. Please make these consistent.

5. Cross-references are mismatched: "the degree of foot rotation (Fig 2)" (line 257) should cite Figure 3 (Figure 2 is flexibility), and "the patient (number 3 in Table 1)" (line 259) should cite Table 3 (Table 1 is the AOFAS rubric).

6. Flexibility terminology is not harmonized: it appears as "rigid/mild/moderate" (Table 3), "no/little/moderate" (Figure 2 legend) and "little or no" (Abstract). The "little to mild" change accepted in revision was not propagated to the Abstract or Figure 2. Please standardize the wording throughout.

7. The forefoot-abduction target is stated as 40 degrees (line 113) but appears as a "30-50 degrees" decision node in Figure 5. Please align the target between text and figure.

8. The pre-treatment tibiocalcaneal angle is reported with a standard deviation larger than its mean (46.75 +/- 52.95 degrees, line 196), which is implausible for an angular measurement and suggests a transcription error to be checked.

9. The pilon impaction fracture is attributed to two feet in the text (patient 3 right, patient 20 left, line 185) but appears documented for patient 3 only in Figure 6. Please confirm and align.

10. Two ethics identifiers appear: the IRB approval number 1.668.854 in the submission metadata, and CAAE 53452216.9.3001.5463 in the manuscript (line 330). If these refer to the same approval, please state both consistently in the Methods.

11. The in-article Data Availability statement (lines 313-316) reads "available from the corresponding author upon reasonable request," which is not consistent with the submission-form statement and the attached S1 Dataset. Please harmonize the wording to reflect that the data are deposited in the Supporting Information.

12. The odds ratio for residual deformity by sex (line 212) is printed as "OR = 20.0; IC 95%, 1;39-301.00": "IC" should read "CI" and "1;39" appears to be a typo for 1.39. A few residual typographical errors remain (e.g., "notfollow" line 192; "s ex" line 23; "similars work" line 309).

13. The 11 feet / 7 patients relationship is correct but reads as contradictory on first pass; a short bridging clause when the surgical subgroup is introduced would help the reader.

14. References: reference 13 (Pirani et al., MRI study) is dated 2021, but the cited volume corresponds to 2001; and reference 17 is attributed in the text to the "American Pediatric Society" (line 85), whereas the source is the American Academy of Pediatrics. Please verify both.

Overall this is a worthwhile study with clear clinical merit. The points above are corrections to existing content rather than requests for new data or analyses, so my recommendation is Minor Revision. I would be glad to look at the revised version.

Reviewer #6: This manuscript reports the treatment outcomes of neglected congenital talipes equinovarus (CTEV) in adults—a topic with limited representation in the literature. While the study addresses an important clinical gap, the presentation is disorganized and lacks the logical flow expected of a scientific paper. My detailed comments are as follows:

Title

• The title suggests that the Ponseti method was the sole treatment modality. However, additional surgical procedures (beyond tendoachilles [TA] tenotomy) were performed. The title should be revised to reflect the full scope of interventions.

Abstract

• As with the title, the abstract states Ponseti as the only treatment objective, yet other procedures were included when correction was incomplete.

• The number of patients (30) does not match the figure reported in the Introduction (43). This inconsistency must be corrected.

• Statistical analysis is inadequately described. The specific correlation test used should be identified, as well as the test applied for pre and post AOFAS score comparisons.

• The conclusion is misleading, as it implies Ponseti alone is sufficient for neglected adult CTEV. It should be clarified to acknowledge the role of adjunctive procedures.

Introduction

• The description of the Ponseti method is overly brief and insufficient, given its central role in the study.

• Key terminology such as “foot flexibility” and “foot rotation” should be introduced here, supported by relevant literature, to justify their use as outcome measures.

Methods

• Patient numbers are inconsistent between the Abstract, Introduction, and flowchart.

• The assessment of foot flexibility appears subjective, with limited validity beyond stratifying subjects into groups. The rationale and limitations should be explained.

• Foot rotation assessment lacks standardization. The methodology for frontal photographs (stance, device used, distance from subject) must be described in detail.

• The TA tenotomy procedure requires clarification: was it open or percutaneous? In adults, Z lengthening is often necessary. Performing the procedure under local anesthesia/sedation alone is unusual and should be justified.

• For all assessments and interventions, the number of surgeons/assessors must be stated, and inter observer reliability should be addressed where appropriate.

• In bilateral cases, the method of data analysis should be specified (e.g., worse foot only, mean/median of both).

• Statistical methods should begin with a normality test, especially given the small sample size. This dictates whether mean or median values are appropriate, and whether parametric or non-parametric tests should be applied.

Results

• Results should begin with a clear description of the study population. Table 3 can summarize this; Table 2 is not useful and may be removed.

• Table 4 does not conform to scientific reporting standards. Analyses should specify the number of subjects (N), the statistical test used (e.g., t test, ANOVA), and p values. For correlation analyses, graphical representation (scatter plots) is more appropriate.

Discussion

• The discussion is superficial and lacks organization.

• Study limitations—many of which are methodological—should be explicitly acknowledged and discussed.

• Greater depth is needed to contextualize findings within existing literature (comparison with neglected cases in children can be used), highlight the challenges of treating neglected adult CTEV, and clarify the role of Ponseti relative to adjunctive procedures.

Reviewer #7: Review of PONE

PONE-D-25-14281

"Treatment of adult neglected clubfoot with Ponseti Method: consecutive case series of thirty-three feet"

Congratulations, it is a very important paper and deserves to be published.

Below are just a few points that can be improved.

Discussion

239 - and the “shortage of qualified surgeons” – this is a dangerous sentence. Lots of surgeons do think that patient could loose walking habilities being so much time without walking, and are afraid to do so, but not because of expertise…

251 – “In our study, for the first time,” – this repeating for the first time is not academically appropriated. This is to be judged by the readers. Please take out these so repeated and inadequate terms.

289 – “stability in neglected clubfoot patients” – It would be nice to take out the neglected term, considered pejorative and negative – better to substitute for non treated adults

It would be nice to have a point in the discussion about bony remodeling, based in what you have seen in the clinical and radiographic aspects of your patients. This is to make a correspondence between Pirani´s 2001 MRI article, this

Nogueira MP, Amaral DT. How much remodelling is possible in a clubfoot treatment? Magnetic resonance imaging study in a 7-year-old child. J Limb Lengthen Reconstr. 2018;4(1):49-54.

And yours.

**********

what does this mean?). If published, this will include your full peer review and any attached files.

If you choose “no”, your identity will remain anonymous but your review may still be made public.

**Do you want your identity to be public for this peer review?**  For information about this choice, including consent withdrawal, please see our Privacy Policy

Reviewer #4: **Yes:** Shokhrukh Erkinov

Reviewer #5: No

Reviewer #6: No

Reviewer #7: No

**********

[NOTE: If reviewer comments were submitted as an attachment file, they will be attached to this email and accessible via the submission site. Please log into your account, locate the manuscript record, and check for the action link "View Attachments". If this link does not appear, there are no attachment files.]

To ensure your figures meet our technical requirements, please review our figure guidelines: https://journals.plos.org/plosone/s/figures

You may also use PLOS’s free figure tool, NAAS, to help you prepare publication quality figures: https://journals.plos.org/plosone/s/figures#loc-tools-for-figure-preparation.

NAAS will assess whether your figures meet our technical requirements by comparing each figure against our figure specifications.

Attachment Submitted filename: Review Adults Ponseti.docx

# Author comment: Author response to Decision Letter 2

**DOI:** 10.1371/journal.pone.0358249.r004

7 Aug 2026

Dear Editor-in-Chief,

Thank you for considering the paper "Treatment of adult neglected clubfoot with Ponseti Method: consecutive case series of thirty-three feet", for publication in the PLos One and for allowing us to re-submit this revised manuscript.

The authors are grateful to the reviewers for the careful appraisal, positive comments, and helpful criticisms. Suggested changes have been addressed. We believe that the quality of the manuscript has been improved and hope that it now meets the quality required for publication in the PLos One.

A detailed point-by-point response is given in the response letter.

In addition, the Abstract has been revised (line 15) to explicitly state that this is the first reported case series of previously untreated adults with neglected clubfoot treated using the Ponseti method.

We are looking forward to your decision regarding the suitability of the revised version of this paper for the Journal.

Thank you very much in advance,

Attachment Submitted filename: Authors response_R2.docx

# Peer review: Decision Letter 2

**DOI:** 10.1371/journal.pone.0358249.r005

**Authors:** Rohan Kothurkar

30 Aug 2026

Treatment of adult neglected clubfoot with Ponseti Method: consecutive case series of thirty-three feet

PONE-D-25-14281R2

Dear Dr. Haje,

We’re pleased to inform you that your manuscript has been judged scientifically suitable for publication and will be formally accepted for publication once it meets all outstanding technical requirements.

Within one week, you’ll receive an e-mail detailing the required amendments. When these have been addressed, you’ll receive a formal acceptance letter and your manuscript will be scheduled for publication.

An invoice will be generated when your article is formally accepted. Please note, if your institution has a publishing partnership with PLOS and your article meets the relevant criteria, all or part of your publication costs will be covered. Please make sure your user information is up-to-date by logging into Editorial Manager at Editorial Manager® and clicking the ‘Update My Information' link at the top of the page. For questions related to billing, please contact billing support.

If your institution or institutions have a press office, please notify them about your upcoming paper to help maximize its impact. If they’ll be preparing press materials, please inform our press team as soon as possible -- no later than 48 hours after receiving the formal acceptance. Your manuscript will remain under strict press embargo until 2 pm Eastern Time on the date of publication. For more information, please contact onepress@plos.org.

Kind regards,

Rohan Kothurkar, PhD

Academic Editor

PLOS One

Additional Editor Comments (optional):

Reviewers' comments:

Reviewer's Responses to Questions

**Comments to the Author**

Reviewer #4: All comments have been addressed

**********

2. Is the manuscript technically sound, and do the data support the conclusions?

Reviewer #4: Yes

**********

3. Has the statistical analysis been performed appropriately and rigorously?

Reviewer #4: Yes

**********

4. Have the authors made all data underlying the findings in their manuscript fully available?

The PLOS Data policy

Reviewer #4: Yes

**********

5. Is the manuscript presented in an intelligible fashion and written in standard English?

Reviewer #4: Yes

**********

Reviewer #4: This manuscript addresses an important and relatively under-studied topic: the use of the Ponseti method in adults with neglected clubfoot. The study is clinically relevant and provides useful information about the effectiveness of this treatment in adult patients.

The main strength of the study is the clear presentation of the treatment process and clinical outcomes. The authors report a considerable improvement in AOFAS scores after treatment, although some patients required additional surgery. The study also mentions physiotherapy after casting, including stretching, strengthening, proprioception, and gait training. However, the rehabilitation protocol is not described in enough detail, particularly regarding the duration and frequency of the exercises.

The relatively small sample size is another limitation and makes it difficult to generalize the findings to all adult clubfoot patients. Longer follow-up and a more detailed rehabilitation protocol could provide stronger evidence in future studies.

In conclusion, this is a useful and clinically relevant study and the manuscript is well organized and the results support the main conclusion. I would recommend publication after minor revisions.

**********

what does this mean?). If published, this will include your full peer review and any attached files.

If you choose “no”, your identity will remain anonymous but your review may still be made public.

**Do you want your identity to be public for this peer review?**  For information about this choice, including consent withdrawal, please see our Privacy Policy

Reviewer #4: No

**********

# Editor report: Acceptance letter

**DOI:** 10.1371/journal.pone.0358249.r006

**Authors:** Rohan Kothurkar

PONE-D-25-14281R2

PLOS One

Dear Dr. Haje,

I'm pleased to inform you that your manuscript has been deemed suitable for publication in PLOS One. Congratulations! Your manuscript is now being handed over to our production team.

At this stage, our production department will prepare your paper for publication. This includes ensuring the following:

* All references, tables, and figures are properly cited

* All relevant supporting information is included in the manuscript submission,

* There are no issues that prevent the paper from being properly typeset

You will receive further instructions from the production team, including instructions on how to review your proof when it is ready. Please keep in mind that we are working through a large volume of accepted articles, so please give us a few days to review your paper and let you know the next and final steps.

Lastly, if your institution or institutions have a press office, please let them know about your upcoming paper now to help maximize its impact. If they'll be preparing press materials, please inform our press team within the next 48 hours. Your manuscript will remain under strict press embargo until 2 pm Eastern Time on the date of publication. For more information, please contact onepress@plos.org.

You will receive an invoice from PLOS for your publication fee after your manuscript has reached the completed accept phase. If you receive an email requesting payment before acceptance or for any other service, this may be a phishing scheme. Learn how to identify phishing emails and protect your accounts at https://explore.plos.org/phishing.

If we can help with anything else, please email us at customercare@plos.org.

Thank you for submitting your work to PLOS One and supporting open access.

Kind regards,

PLOS One Editorial Office Staff

on behalf of

Dr. Rohan Kothurkar

Academic Editor

PLOS One
